# Undercarboxylated OCN Inhibits Chondrocyte Hypertrophy and Osteoarthritis Development through GPRC6A/HIF-1α Cascade

**DOI:** 10.7150/ijbs.105560

**Published:** 2025-06-23

**Authors:** Zhangzhen Du, Yongqi Zhao, Ke Zhang, Qiaozhen Qin, Changyi Luo, Jiamei Wu, Heyang Zhang, Shuirong Liu, Zhenhua Xu, Jing Zheng, Shuli Fan, Xiaoxia Jiang, Xu Li, Yan Wang

**Affiliations:** 1 Beijing Institute of Basic Medical Sciences, 27 Taiping Road, Beijing 100850, P.R. China.; 2 The First Hospital of China Medical University, Shenyang 110000, P.R. China.; 3 Anhui Medical University, Hefei 230032, P.R. China.; 4 Chengdu Fifth People's Hospital, Chengdu 610000, P.R. China.

**Keywords:** OCN, ucOCN, Osteoarthritis, GPRC6A, HIF-1α

## Abstract

Initial investigations established osteocalcin (OCN) as a pivotal factor in bone formation. Fully carboxylated osteocalcin (cOCN) exhibits a high affinity for hydroxyapatite within the bone matrix, yet under specific physiological conditions, it may undergo decarboxylation, thereby acquiring endocrine regulatory capabilities. Recent findings suggest a potential protective role for undercarboxylated osteocalcin (ucOCN) beyond bone, influencing various systems, including the brain, pancreas, muscle, and gonads, where its effects are well established. Although increased intracellular OCN expression is often considered a marker of osteoarthritis (OA) and chondrocyte hypertrophy, the specific role of extracellular ucOCN in chondrocytes remains largely unexplored and has received little attention, especially regarding its potential to modulate OA-related changes. This study used OCN knockout (OCN^-/-^) mice and found that OCN absence increased collagen type X (COL10) and matrix metalloproteinase 13 (MMP13) expression in chondrocytes, despite a lack of severe OA phenotype. A declining trend of ucOCN in synovial fluid was observed in arthritis models and OA patients, suggesting a role in OA progression. Elevation of ucOCN levels led to the downregulation of COL10a1 and MMP13 expression, accompanied by a marked improvement in cartilage integrity in murine models of arthritis. Additionally, ucOCN regulated the G protein-coupled receptor class C group 6 member A (GPRC6 A) and Hypoxia-inducible factor 1-alpha (HIF-1α) pathways, promoting TIMP3 expression and autophagy in chondrocytes, indicating distinct molecular mechanisms behind its protective effects.

## Introduction

Osteoarthritis (OA) is the most common chronic joint disease in individuals over 65, characterized by structural, compositional, and functional cartilage changes 1-3. Although genetic factors, biomechanical stress, and biological influences like trauma and infection contribute to OA progression 4, 5, its exact etiology remains unclear. Joint homeostasis relies on tightly regulated signaling pathways, with chondrocyte phenotype maintenance being a key indicator of this balance 6-9. Under physiological conditions, articular chondrocytes arrested at a pre-hypertrophic stage with both low metabolic and regenerative ability, and there is a delicate balance between chondrocytes and extracellular matrix 10. In OA, chondrocytes undergo hypertrophic changes, expressing markers such as COL10, MMPs and a disintegrin and metalloproteinase with thrombospondin motifs (ADAMTSs), which drive cartilage degradation 9, 11.

OA is a whole joint disease, affecting all components of the joint 10, 12-14. Extensive research has demonstrated the crosstalk within the skeletal muscle system, particularly highlighting the bioactive role of the bone-derived hormone OCN and its involvement in these regulatory processes 13-16. OCN signaling is essential for maintaining muscle mass, enhancing exercise adaptability, and promoting tissue repair, particularly in tendon sheaths 16-19. Furthermore, OCN has been shown to positively regulate various age-related physiological functions, including memory, male fertility, and muscle adaptability 17. These findings raise the question of whether OCN exerts similar regulatory effects in the context of another age-related musculoskeletal disorder, osteoarthritis. OCN is predominantly synthesized by osteoblasts 20, 21. Depending on its degree of carboxylation, mature OCN is typically classified into two subtypes: γ-carboxylated OCN (cOCN) and undercarboxylated OCN (ucOCN). Fully carboxylated OCN primarily deposits within the bone extracellular matrix due to its strong affinity for hydroxyapatite, and it can be decarboxylated into ucOCN by osteoclasts 22-24, subsequently entering the circulatory system. Although both ucOCN and cOCN are detectable in the circulation, the majority of studies have established that only ucOCN exhibits biological endocrine activity 22, 23, thus earning its designation as bioactive OCN. Given that OCN is secreted during the maturation phase of osteoblasts and highly expressed in OA chondrocytes 6, 10, it has long been regarded as a marker of hypertrophic chondrocytes. However, whether ucOCN exerts effects analogous to those of OCN in OA pathogenesis remains to be elucidated.

We therefore initiated an investigation into the functional role of ucOCN in chondrocytes. Chondrocytes from both neonatal and adult OCN^-/-^ mice displayed a more pronounced hypertrophic phenotype, which contrasts with previous reports identifying OCN as a hypertrophy marker. However, in an adult arthritis model, OCN knockout did not exacerbate the arthritis phenotype. This observation led us to hypothesize that OCN's role in cartilage homeostasis may vary depending on its form, particularly the undercarboxylated form-ucOCN. To test this, we manipulated ucOCN levels in cell supernatants and synovial fluid in a series of *in vivo* and *in vitro* experiments. Our results showed that ucOCN inhibits chondrocyte hypertrophy and protects against osteoarthritis, primarily through the activation of the HIF-1α pathway via the GPRC6A receptor.

## Materials and Methods

### Animal Studies

All procedures for animal use were in accordance with the national guidelines for the use of animals in scientific research. Additional approval was granted by the Administrative Panel on Laboratory Animal Care at the Beijing Institute of Basic Medical Sciences. All mice were maintained on a pure C57BL/6NCrSlc genetic background. The OCN^-/-^ mice were obtained from Beijing BIOCYTOGEN Gene Biotechnology Co., Ltd. for model organisms. All mice were maintained in a specific pathogen-free environment with a 12-hour light/12-hour dark cycle and provided ad libitum with water and chow.

### Collection of Human Tissue Samples

All human tissue samples were sourced from patients at the First Affiliated Hospital of China Medical University, with informed consent obtained from the patients. The articular cartilage was obtained from patients who underwent total knee arthroplasty for OA and their synovial fluid was collected at the time of surgery, these patients were excluded from systemic metabolic diseases such as diabetes and rheumatoid diseases. They were all female patients aged between 65-70 years. The synovial fluid in the control group was obtained from young surgical patients with meniscal injuries aged 30-35 years. These patients had meniscal injuries of 1-3 months duration, no metabolic diseases, no intraoperative destruction of articular cartilage, and no significant blood mixing in the joint fluid.

### Primary Chondrocytes Isolation and Culture

Primary chondrocytes were isolated from 5 days old mice according to established protocol 25. Briefly, mice were sacrificed with an overdose of anesthetic and immersed in 75% ethanol for 5 minutes. In sterile flow hood, the femoral head, tibial plateau and femoral condyles of mice were separated under anatomical microscope, the surrounding soft tissue was removed as clean as possible, digested with 0.02% type I collagenase (BioFroxx) for 20 minutes after rinsing twice with sterile PBS, then the soft tissue was removed again. The cartilage was rinsed twice with sterile PBS again and then digested with 0.02% type II collagenase (BioFroxx) for about 5 hours to single cell suspension at 37°C in a thermal incubator under 5% CO_2_. The isolated cells were seeded into culture dish and cultured with a DMEM: F12=1:1 medium (Sigma) contained 10% FBS (sigma) and 1% penicillin-streptomycin. The cells were passaged when fused to 80% and the P1 cells were used for subsequent cell experiments.

### Micromass Culture

Micromass cultures were taken as described previously 9. Briefly, 5×10^5^ primary chondrocytes were centrifuged at 300 × g for 10 minutes. After 3 days culturing in the same medium in the centrifuge tubes, the resulting cell pellets were transferred into a low-attachment petri dish and incubated in the same medium for 21 days.

### Quantitative Real-time PCR (qRT-PCR)

Cells in a six-well plate were washed twice with 2 ml cold PBS, followed by addition of 1 mL of TRIzol. Cells were incubated for 5 minutes at room temperature and collected in Eppendorf tubes. Micromass or tissue samples were frozen in liquid nitrogen, crushed by a multibead shocker and immediately extracted for RNA. For the real-time RT-PCR analyses, 500 ng of total RNA was reverse-transcribed into first-strand cDNA by using ReverTra Ace (Toyobo). PCR amplification was performed in a reaction volume of 20 μL containing 1 μL cDNA and 4 μL SYBR FAST qPCR Master Mix (Tsingke). The primer sequences used for qRT-PCR were shown in supplementary Table 1. Data were analyzed via comparison Ct (2^-ΔΔCt^) method and expressed as fold change compared with β-actin. Each sample was analyzed in triplicate.

### Protein Extraction

To detect protein expression, the sample was washed twice with cold PBS, and 100 μL RIPA lysis buffer (Solarbio) premixed with protease inhibitors was used to lysis the whole cell after removing PBS, cell lysates were collected after lysing for 30 minutes on ice while ensuring that lysates were collected as much as possible. After centrifuged at 12,000 × g for 15 minutes at 4°C, the supernatant was collected and protein solution was then mixed with loading buffer (Sango) and boiled at 95°C for 5 minutes. Samples were stored at -80°C.

### Western Blotting

The pre-prepared samples were separated on sodium dodecyl sulfate-polyacrylamide gels (SDS-PAGE) and transferred to a PVDF membrane (Civita). After blocking in 5% skim milk for 1 hour at room temperature, the membrane were cultured with following antibody: anti-MMP13 (Abclonal, A16920, 1:1000), anti-COL10a1 (Abclonal, A11645, 1:1000), anti-OCN (Santa Cruz, sc-390877, 1:500), anti-COL2a1(Abclonal, A1560 , 1:1000), anti-GPRC6A (Solarbio, K005896P, 1:1000), anti-HIF-1α (Affinity, AF1009, 1:1000), anti-TIMP3 (Affinity, DF6432, 1:1000), anti-Phospho-AKT (Cell Signaling Technology, 4060, 1:1000), anti-Phospho-mTOR (Cell Signaling Technology, 5536, 1:1000), anti-Phospho-P70S6 Kinase (Cell Signaling Technology, 9205, 1:1000), anti-Phospho-4EBP1 (Abclonal, AP0030, 1:1000), anti-P62 (Cell Signaling Technology, 16177, 1:1000), anti-LC3b (Cell Signaling Technology, 3868, 1:1000) at 4°C overnight. The membranes were washed with TBST then incubated with HRP-conjugated secondary antibody (Abclonal) at room temperature for 1 hour. The membrane then got washed 3 times by TBST, then exposed to ECL solutions (Applygen) for 5-10 minutes and developed by Hyper film ECL.

### Destabilized Medial Meniscus (DMM) Surgery

12-weeks-old male mice were injected 1.25% Avertin intraperitoneally for anesthesia according to the dosage of 0.2 mL/kg. The fur around knee joints were shaved gently and disinfected with 2% iodophor. A 5 mm size incision was performed along the medial edge of the patellar ligament, expose the knee joint cavity layer by layer. The anterior horn of the medial meniscus was dissociated from tibial plateau under the anatomical microscope with microsurgical instruments. The muscle and skin around knee joint were sutured with 4-0 silk sutures. The sham operation group only exposed the knee joint cavity and sutured as the DMM group without any ligament transection. The mice were sacrificed at the designed time points and the knee joints and synovial fluid were collected.

### Safranin O and Fast Green Staining

The knee joint samples were decalcified in EDTA decalcification solution (Solarbio) for about 3 weeks and the solution was changed every week until no obvious resistance to needling. Then the samples were dehydrated, embedded and sliced into 5 μm paraffin sections. The sections were dewaxed twice in xylene for 10 minutes each, and then rehydrated in 100% alcohol, 95% alcohol and 70% alcohol for 5 minutes successively. Then stained in Weigert's Iron Hematoxylin for 5 minutes and gently wash the excess pigment with flowing water and differentiated in 1% acid-alcohol for 5 seconds. The sections were rinsed gently in flowing water and stained with 0.2% Fast Green for 3 minutes, then rinsed quickly with 1% acetic acid solution for no more than 30 seconds and stained in 0.1% safranin O solution for 10 minutes. Subsequently, the sections were dehydrated using a graded alcohol series for hydration, cleared with xylene twice for 2 minutes each, and subsequently mounted with a resinous medium.

### Hematoxylin and Eosin (H&E) Staining

The paraffin sections were subjected deparaffinized, re-hydrated as described above. The sections were stained with hematoxylin solution for 5 minutes, then washed in flowing water for 10 minutes. The sections were differentiated by 1% acid alcohol for 30 seconds, then washed in flowing water for 3 minutes. The sections were stained in 0.2% ammonia water for 30 seconds and washed in flowing water for 5 minutes. The sections were counterstained in eosin-phloxine solution for 1 minute and washed in flowing water for 5 minutes. The sections dehydrated, cleaned and mounted as described above. Synovium score was evaluated according to the reported method 12.

### Histology and Immunohistochemistry (IHC)

The paraffin sections were subjected deparaffinized, re-hydrated as described above. Antigen repair was performed with sodium citrate solution at 95°C for 10 minutes and rinsed three times with PBS. Soak the slices in 3% H_2_O_2_ for 10 minutes to remove endogenous peroxidase. The sections were washed twice with PBS, then immersed in blocking buffer containing 0.2% TritonX-100, 3% bovine serum albumin and 5% goat serum for 1 hour, and incubated in 4°C overnight with primary antibody solution. The sections were washed with PBS for 5 times, each time for 5 minutes, and stained with secondary antibody at room temperature for 1 hour. After washed with PBS for 5 times, each time for 5 minutes, the sections were stained in Weigert's Iron Hematoxylin for 5 minutes and gently wash the excess pigment with flowing water and differentiated in 1% acid-alcohol for 5 seconds. Finally, the sections were dehydrated, cleaned and mounted as described above. Images were obtained by a FluoView FV1000 confocal microscope (Olympus). Proportion of positive cells or mean gray value was measured for statistical analysis. The primary antibodies used were as follows: anti-MMP13 Affinity, AF5355, 1:400), anti-COL10a1 (Affinity, DF13756, 1:400), anti-OCN (Santa Cruz, sc-390877, 1:200), anti-COL2a1(Abclonal, A1560 1:200), anti-GPRC6A (Solarbio, K005896P, 1:400), anti-HIF-1α (Affinity, AF1009, 1:400), anti-TIMP3 (Affinity, DF6432, 1:400), anti-Phospho-mTOR (Cell Signaling Technology , 5536, 1:400).

### Immunofluorescent (IF) Staining

The frozen sections of the lower limbs from newborn mice were washed twice with PBS. They were then blocked with a blocking buffer containing 0.2% Triton X-100, 3% bovine serum albumin, and 5% goat serum for 1 hour. Following blocking, the sections were immunostained with the primary antibody solution at 4°C overnight. After incubation, the sections were washed five times with PBS, each wash lasting 5 minutes. Subsequently, the sections were stained with the secondary antibody at room temperature for 1 hour. Finally, the sections were mounted with a sealing agent containing DAPI (Slolabio). The primary antibodies used are as follows: anti-MMP13 (Affinity, AF5355, 1:200), anti-COL10a1 (Affinity, DF13756, 1:200), anti-HIF-1α (Affinity, AF1009, 1:200), anti-COL2a1 (Affinity, AF0135, 1:200).

### OA Evaluation

Cartilage destruction was scored using the well-known established OARSI (Osteoarthritis Research Society International) scoring system 9, 11, 26 based on the Safranin O and Fast green stained sections. OARSI scoring involved selecting four out of eight consecutive sections to represent the weight-bearing areas of the femur and tibia. The two lowest scores were averaged, and the sum of the femoral and tibial scores was designated as the OARSI score for each knee. OARSI scoring was performed by two independent, double-blinded colleagues.

### Collection of Mouse Knee Joint Synovial Fluid

Mouse patellar ligament was exposed under anesthesia, and gently cut the patellar ligament with a syringe needle. Articular cavity was washed by 150 μL sterile PBS, and the assistant absorbs the lavage solution along the other side of the incision. Lavage fluid was used for ELISA test.

### Enzyme-Linked Immunosorbent Assay (ELISA)

Lavage fluid from the joint cavity was directly used for ELISA tests. After initial collection, the lavage fluid was centrifuged at 12,000 × g for 5 minutes at 4°C to eliminate cell debris, and the supernatant was collected. The concentrations of total OCN and ucOCN were determined using ELISA kits according to the manufacturer's instructions: for humans-ucOCN (Qingyun Biology, MM-50504H1), cOCN (Qingyun Biology, MM-50505H1), and total OCN (Qingyun Biology, MM-50709H1); for mice-total OCN (Jonlnbio, JL20366) and ucOCN (Jonlnbio, JL20562). The content of mouse cOCN was calculated by the difference between total OCN and ucOCN.

### Application of Recombinant ucOCN, Recombinant cOCN and OCN Antibody *in vitro* and *in vivo*

For the *in vitro* cell experiment, approximately 3.5×10^5^ cells were seeded per well of the 6-well cell culture plate. After two days of inoculation and incubation, the levels of uncarboxylated osteocalcin (ucOCN) and carboxylated osteocalcin (cOCN) detected in the supernatant were used as a reference for subsequent experiments. In the subsequent experiments, the inoculated cells remained basically the same. 5 ng/mL Recombinant ucOCN (Abcam, ab274873) and Recombinant cOCN (AnaSpec AS-22830) were added into the supernatant of pretreated chondrocytes, and samples were collected for qRT-PCR and WB at 24 hours. DMM operations were performed on the right knee joints of C57BL/6NCrSlc male mice at 12 weeks of age. Mouse IgG (CON) and ucOCN was diluted with PBS to 0.5 ng/μL and OCN antibody (Santa Cruz, sc-390877) was diluted with PBS to 1.5:10. 10 μL of the diluted reagent was injected into the intra-articular space of the knee joints twice a week. After 7 weeks of injection, mice were sacrificed, and knee joints were collected for histological analysis.

### Co-immunoprecipitation

Mouse primary chondrocytes treated with 5 ng/mL recombinant ucOCN for 4 hours were lysed with cold IP-lysis buffer (20 mM Tris-HCl (pH 7.4), 0.15 M NaCl, 1 mM EDTA, 1 mM EGTA, 1% Triton X-100 with protease inhibitors cocktail (Cell Signaling Technology) and 500 mM Dithiothreitol (DTT) for 2 hours and centrifuged at 15, 000 × g for 30 minutes at 4°C. Supernatant of whole cell lysates were transferred into new tubes. The protein incubated with anti-OCN antibody (Santa Cruz, sc-390877) or IgG (for control) for overnight at 4°C, then mixed with agarose A/G for 2 hours. The precipitant was collected and washed 3 times with IP-lysis buffer, dissolved in sample loading buffer and heat denaturing with 4× laemmli sample buffer (Omiget) and subsequently subjected to standard western blotting analysis.

### Gelatin Zymography

The collected supernatant from monolayer cultured cells was concentrated by ultrafiltration and normalized to the same concentration. Gelatin zymography was taken as reported protocol 27. Briefly, 20 μL concentrated supernatant were loaded into the wells of prepared gels (10% polyacrylamide gels containing 0.1% gelatin).

After electrophoresis, the gel was washed with a buffer containing 2.5% Triton X-100, 50 mM Tris-HCl, 5 mM CaCl₂, and 1 μM ZnCl₂. Subsequently, it was incubated in a shaking bath (37°C) for 24 hours with a buffer containing 1% Triton X-100, 50 mM Tris-HCl, 5 mM CaCl₂, and 1 μM ZnCl₂. Finally, the gel was stained with Coomassie Brilliant Blue (1% solution in 10% acetic acid, 10% isopropyl alcohol, diluted with ddH₂O).

### RNA-Seq Analysis

Primary chondrocytes were treated with 5 ng/mL recombinant ucOCN or mouse IgG (CON) as control for 24 hours in an inflammatory environment 20 ng/mL IL-1β and serum free medium. RNA was extracted with a standard procedure. RNA quality was tested by a Nanodrop 2000 spectrophotometer (Thermo Scientific). RNA samples were sent for library preparation and sequencing on the Illumina NovaSeq 6000 platform by Shanghai Majorbio Bio-Pharm Technology. The data were analyzed on the free online platform of Majorbio I-Sanger Cloud Platform (www.i-sanger.com) or using R software.

### Measurement of Glycosaminoglycan Release and Collagen Degradation in Cartilage Explants

As previously described in the literature, glycosaminoglycan (GAG) release was quantified using the 1,9-dimethylmethylene blue (DMMB) assay, and collagen degradation was assessed by measuring the levels of C-terminal telopeptide of type II collagen (CTX-II) via ELISA in cartilage explants 28. Femoral head cartilage was isolated from four male OCN^-/-^ mice (3 weeks old, 2 hips per mouse) for each group. The cartilage explants were pooled and distributed into serum-free medium, with four replicates established per group. Explants were transferred to 6-well plates and treated with or without 10 ng/mL murine TNF-α (PeproTech) and 20 ng/mL murine IL-1β (PeproTech), in the presence of either 5 ng/mL ucOCN or 5 ng/mL cOCN. After 72 hours, the GAG release in the supernatant was quantified using the DMMB assay (GENMED, GMS19239.4), with chondroitin sulfate (Sigma, C4384) as a standard. After collection, femoral heads were weighed and fixed for future use. GAG release was expressed as the ratio of released GAG amount to femoral head weight (µg/mg tissue).

For collagen degradation assessment, explants were stimulated with 10 ng/mL TNF-α and 20 ng/mL IL-1β, in the presence of either 5 ng/mL ucOCN or 5 ng/mL cOCN, with medium replenished every 3 days, for a total of 10 days. CTX-II levels in the supernatant were measured using a CTX-II ELISA kit (FineTest, EM0960), following the manufacturer's protocol.

### Statistics

Data were expressed as averages and standard deviations. For the comparison between two groups, we carried out unpaired two-tailed Student's *t*-test. For three or more group, one-way ANOVA or two-way ANOVA was used with Tukey *post hoc* test. *p* < 0.05 was considered as statistically significant. GraphPad Prism software (Version 7.0) was used for statistical analysis.

## Results

### OCN Deficiency Leads to Increased Chondrocyte Hypertrophy

**Figure 1:**
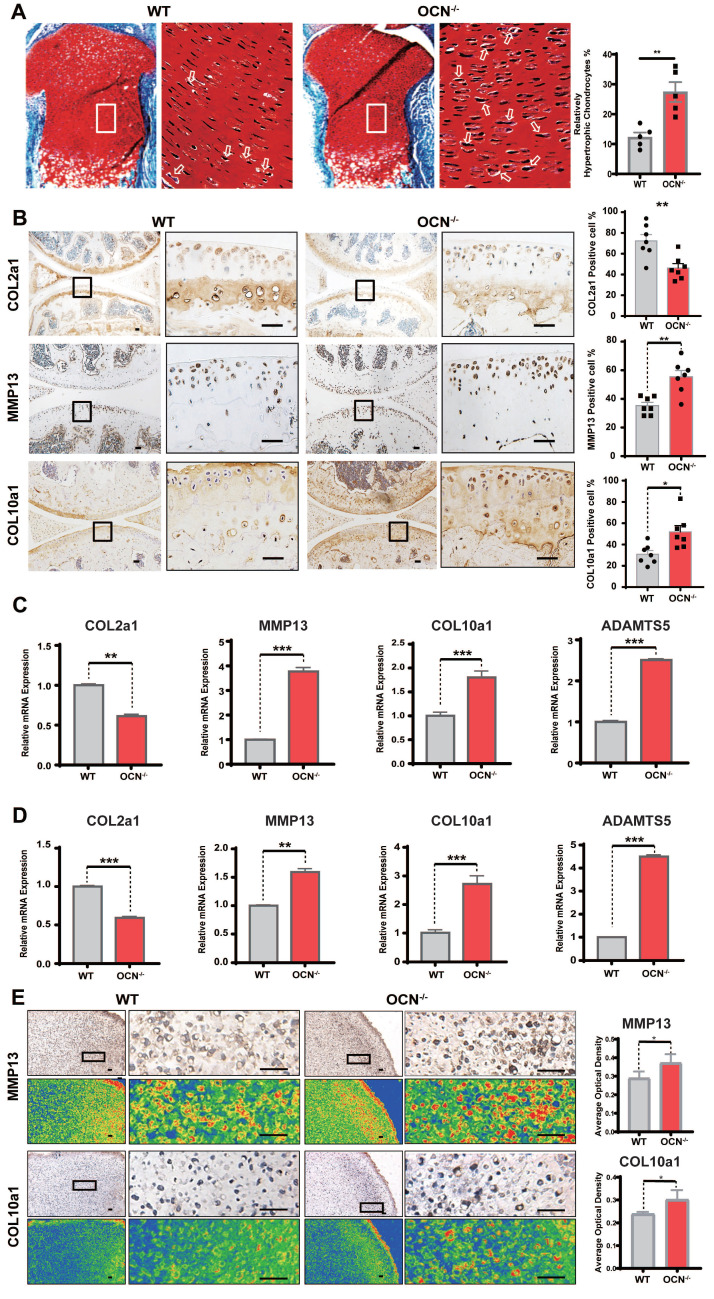
OCN deficiency leads to increased chondrocyte hypertrophy**.** (A) Safranin O and fast green staining of representative paraffin sections of femora of the newborn WT mice. The white boxes depict regions of higher magnification of the hypertrophic zone of the growth plate as shown on the right. The arrows depict the hypertrophic chondrocytes. Statistical analysis of the proportion of relatively hypertrophic chondrocytes is shown on the right. (n = 5 mice per group). (B) Immunohistochemistry (IHC) staining of representative paraffin sections of COL2a1, MMP13 and COL10a1 expression in articular cartilage of WT mice and OCN^-/-^ mice. The black boxes depict regions of higher magnification. Statistical analysis is on the right. (n=7 mice per group). (C) Gene expression analysis of hypertrophic markers of primary chondrocytes isolated from WT and OCN^-/-^ newborn mice after monolayer culture. (D) Gene expression analysis of hypertrophic markers of primary chondrocytes isolated from WT and OCN^-/-^ newborn mice after 3D micromass culture for 21 days. (E) IHC staining of representative paraffin sections of MMP13 and COL10a1 expression in chondrocytes of WT and OCN^-/-^ newborn mice after 3D micromass culture for 21 days. The black boxes depict regions of higher magnification. Grade map visualization displayed by the Slide Viewer software is shown below, red represents the intensity of staining (n=3 per group). Statistical analysis is on the right. Scale bar, 100 μm. WT, wild type. OCN^-/-^, OCN knockout. Student's t-test for two groups, one-way ANOVA for three or more. *p < 0.05. ** *p* < 0.01. *** *p* < 0.001.

The impact of OCN on mouse cartilage was assessed using OCN^-/-^ mice, with both Bglap and Bglap2 genes deleted under the C57BL/6NCrSlc genetic background (Figure S1). We first confirmed the knockout efficacy, showing a significant reduction in OCN expression in chondrocytes (Figure S2A, B). Safranin O and fast green staining revealed no notable changes in growth plate thickness post-knockout, consistent with reports of no limb deformities in OCN^-/-^ mice 24. However, OCN^-/-^ mice exhibited more pronounced chondrocyte hypertrophy in the growth plate compared to WT mice, characterized by increased cell volume, enlarged nuclei, and more abundant cytoplasm 9 (Figure 1A). H&E staining showed no significant morphological differences in articular cartilage (Figure S2C), which may be due to the more active state of growth plate chondrocytes, as OCN's effect is less evident in quiescent chondrocytes.

IHC results of adult mouse knee joints revealed that, compared to WT, OCN^-/-^ mice had decreased COL2a1 expression and increased MMP13 and COL10a1 expression (Figure 1B), indicating a tendency toward chondrocyte hypertrophy following OCN deletion. We further assessed mRNA levels of COL2a1, MMP13, COL10a1, and ADAMTS5 in WT and OCN^-/-^ chondrocytes from newborn mice. qRT-PCR showed significant upregulation of MMP13, COL10a1, and ADAMTS5, and a marked reduction in COL2a1 in both monolayer and 3D micromass cultures of OCN^-/-^ chondrocytes (Figure 1C, D). IHC staining of micromass samples confirmed elevated COL10a1 and MMP13 expression in OCN^-/-^ chondrocytes (Figure 1E, S2D). Collectively, these results suggest that OCN inhibits chondrocyte hypertrophy both *in vivo* and *in vitro*.

### Undercarboxylated OCN Decreases in Knee Synovial Fluid During the Progression of OA

**Figure 2:**
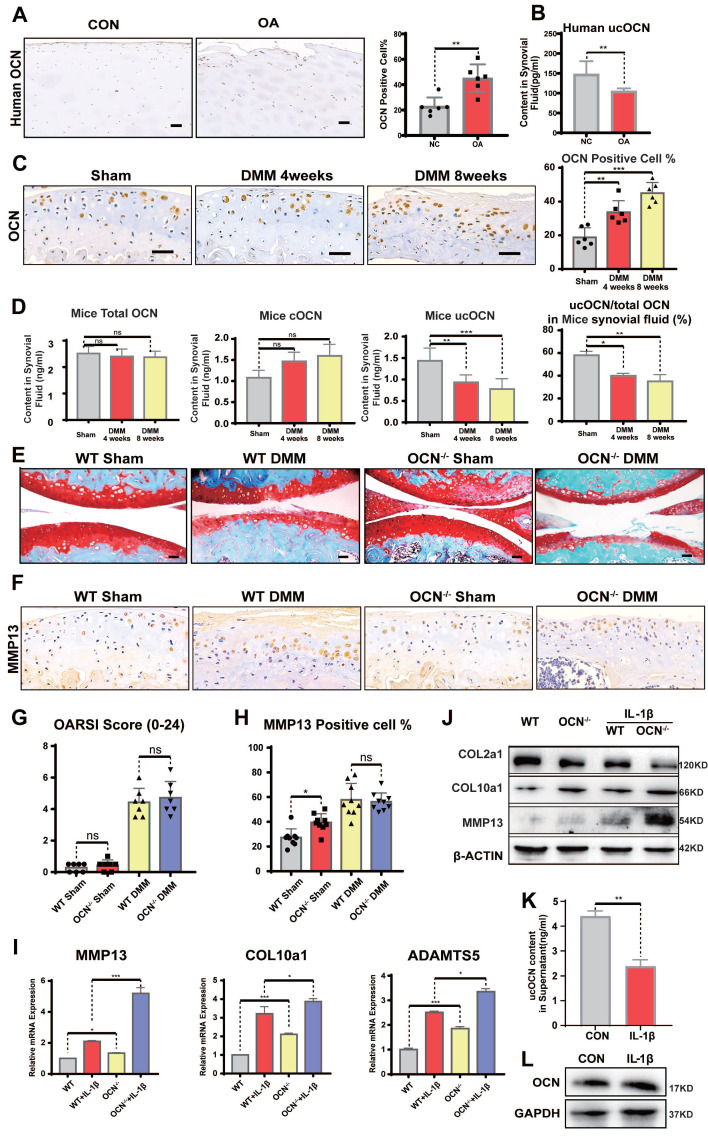
Undercarboxylated OCN expression is reduced in WT mice synovial fluid and OCN^-/-^ mice do not develop more severe OA than WT mice after DMM surgery. (A) IHC staining of representative paraffin sections of OCN expression in articular cartilage of human LFC and MFC with OA (n=6 mice per group). (B) ELISA analysis of ucOCN levels in Human SF. (n=12, CON group was obtained from young surgical patients with meniscal injuries aged 30-35 years). (C) IHC staining of representative paraffin sections of OCN expression in articular cartilage of WT mice 1 and 2 months after DMM surgery. (n=6 mice per group). (D) ELISA analysis of total OCN and ucOCN during DMM-induced OA progression in WT mice. (n=6 mice for Sham and 4 weeks. n = 4 for 8 weeks). (E, G) Safranin O and fast green staining of representative paraffin sections of knee joint of OCN^-/-^ and WT mice 2 month after DMM surgery and OARSI score. (n=7 mice per group). (F, H) IHC staining of representative paraffin sections of MMP13 expression in articular cartilage of OCN^-/-^ and WT mice 2 month after DMM surgery. (n=9 mice per group). (I) Gene expression analysis of hypertrophic markers of primary chondrocytes isolated from WT and OCN^-/-^ mice with or without IL-1β (20 ng/mL) for 24 hours. (J) Western blotting analysis of COL2a1, COL10a1 and MMP13 of primary chondrocytes isolated WT and OCN^-/-^ mice with or without IL-1β (20 ng/mL) for 24 hours. (K) ELISA analysis of ucOCN content of supernatant of chondrocytes from WT mice treated with or without 20 ng/mL IL-1β for 24 hours. (L) Western blotting analysis of the intracellular OCN of WT primary chondrocytes treated with IL-1β (20 ng/mL) for 24 hours. Scale bar, 100 μm. WT, wild type. OCN^-/-^, OCN knockout. Student's t-test for two groups, one-way ANOVA for three or more. *p < 0.05. **p < 0.01. ***p < 0.001. Cells were maintained under monolayer culture conditions.

To investigate the role of OCN in articular cartilage homeostasis, we first examined OCN expression in human tissue samples. Lateral femoral condyles (LFC) from OA patients with relatively intact cartilage served as controls, while medial femoral condyles (MFC), showing significant degeneration, represented the OA group. Consistent with previous studies, IHC revealed a significant increase in OCN expression in MFC chondrocytes (Figure 2A). We also measured OCN levels in the synovial fluid (SF) of OA patients. Compared to younger patients with meniscal injuries, older OA patients showed no significant differences in cOCN or total OCN (tOCN) (Figure S2E), but a marked decrease in undercarboxylated OCN (ucOCN) (Figure 2B).

Next, we performed DMM surgery on WT and OCN^-/-^ mice. In WT DMM mice, OCN expression in chondrocytes, as well as tOCN, cOCN, and ucOCN levels in SF, mirrored the findings in human samples when compared to sham-operated mice (Figure 2C, D). These results suggest that OCN contributes to maintaining cartilage integrity. The divergent trends of intracellular OCN and extracellular ucOCN suggest they may have distinct roles in OA development.

### OCN^-/-^ Mice Do Not Develop More Severe OA than WT Mice after DMM Surgery

When analyzing OA phenotypes, the purported role of OCN in inhibiting chondrocyte hypertrophy appeared diminished. OCN^-/-^ DMM mice showed no significant differences in OA characteristics, including OARSI scores (Figure 2E, G) and MMP13 expression (Figure 2F, H), compared to age-matched WT mice. This unexpected result caught our attention, as experiments in non-operated and sham-operated adult mice demonstrated OCN's role in suppressing chondrocyte hypertrophy markers. Interestingly, despite showing similar levels of joint destruction, these results suggested a more rapid upregulation in hypertrophy-related protein expression in WT mice compared to OCN^-/-^ mice in the OA model.

To further investigate OCN's effect on OA chondrocytes, we isolated primary chondrocytes from WT and OCN^-/-^ mice and treated them with 20 ng/ml IL-1β for 24 hours to simulate an OA environment *in vitro*. Contrary to the *in vivo* results, IL-1β-treated OCN^-/-^ chondrocytes showed significant upregulation of OA markers, including MMP13, COL10a1, ADAMTS5, IL-6, and ALP (Figure 2I, S2F). Protein analysis revealed increased MMP13 and COL10a1, along with reduced COL2a1 levels (Figure 2J). These findings suggest that OCN may function as a protective barrier against osteoarthritis progression.

Given OCN's well-documented endocrine role and its diverse effects in different carboxylated forms, as reported in other biological systems, we hypothesize that its influence on chondrocytes arises from a combination of intracellular OCN and secreted ucOCN. Intracellular OCN appears to be detrimental, as previously reported 21, 24, 25, while secreted ucOCN seems to offer protection. Our data support this hypothesis: in non-operated and sham-operated mice, ucOCN in the joint cavity had a protective effect, as evidenced by more pronounced hypertrophy following OCN knockout. During OA progression, OCN expression in chondrocytes increased, whereas ucOCN levels in the synovial fluid decreased, accelerating hypertrophy. At the cellular level, 24 hours after IL-1β stimulation, ucOCN levels in the supernatant significantly decreased (Figure 2K), while intracellular OCN levels showed no noticeable change (Figure 2L), reinforcing the notion of a protective role for ucOCN.

### Secreted ucOCN Protects Chondrocyte from Hypertrophy *in vitro*

**Figure 3:**
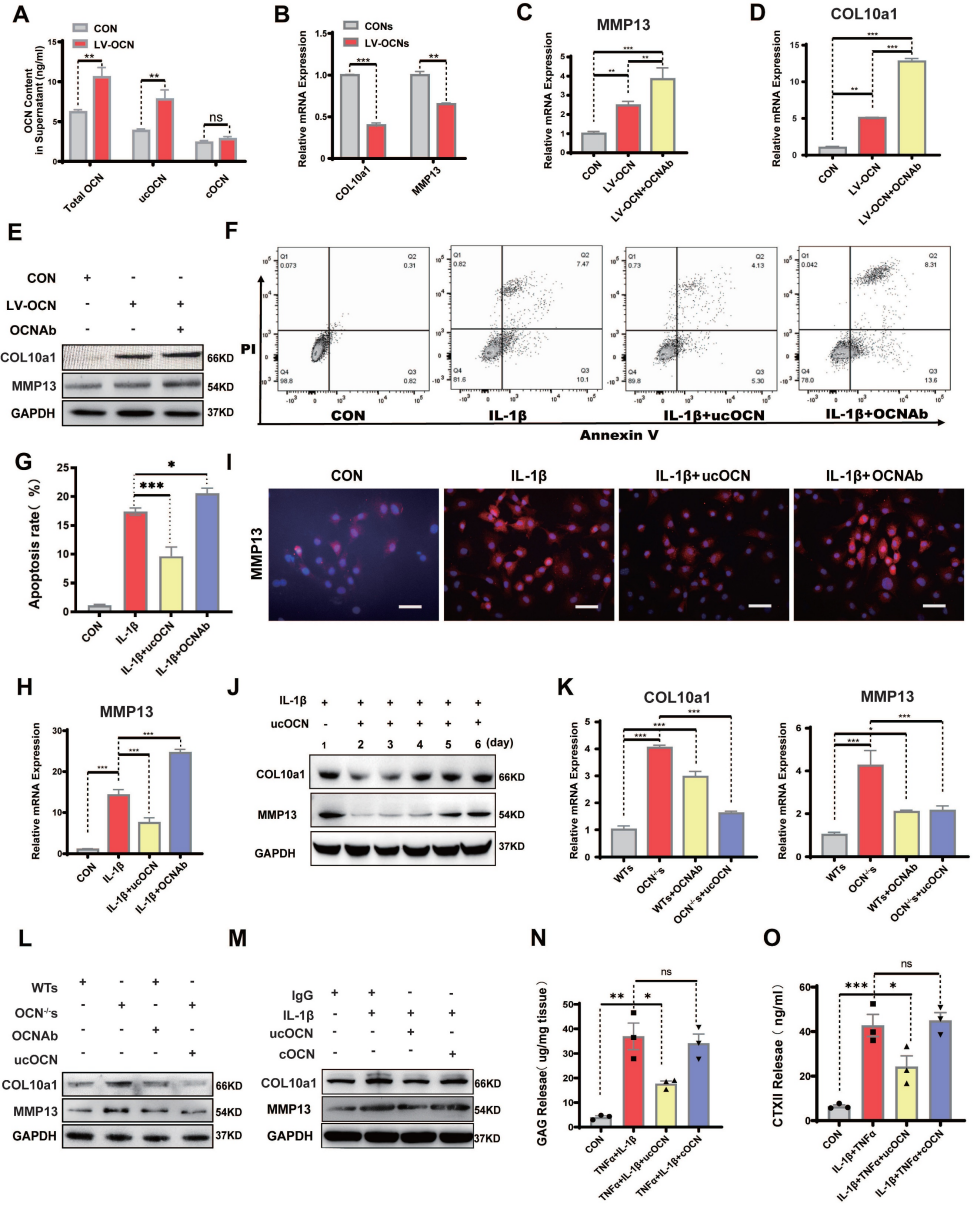
Secreted ucOCN protects chondrocyte from hypertrophy *in vitro.* (A) ELISA analysis of total OCN, ucOCN, cOCN of supernatant harvested from WT chondrocytes treated with control (CON) or OCN-overexpressing lentivirus (LV-OCN). (B) Gene expression analysis of hypertrophic markers of WT chondrocytes treated with supernatant harvested from WT chondrocytes treated with control (CONs) or OCN-overexpressing lentivirus (LV-OCNs) for 24 hours. (C-E) Gene expression and western blotting analysis of MMP13 and COL10a1 of WT primary chondrocytes treated with LV-control (CON), LV-OCN, LV-OCN + OCN antibody respectively for 24 hours. (F, G) Flow cytometric analysis of apoptosis of WT chondrocytes treated with mouse IgG (CON), IL-1β, IL-1β + recombinant ucOCN, IL-1β + OCN antibody respectively for 24 hours. (H, I) Gene and IF analysis of MMP13 of WT chondrocytes treated with mouse IgG (CON), IL-1β, IL-1β + recombinant ucOCN, IL-1β + OCN antibody respectively for 24 hours. (J) Western blotting analysis of MMP13 and COL10a1 of WT chondrocytes treated with ucOCN at different time point (1-6 day). (K, L) Gene expression and western blotting analysis of MMP13 and COL10a1 of OCN^-/-^ primary chondrocytes treated with WT chondrocytes supernatant (WTs), OCN^-/-^ chondrocytes supernatant (OCN^-/-^ s), WTs with OCN antibody, OCN^-/-^s with 5 ng/mL recombinant ucOCN respectively for 24 hours. (M) Western blotting analysis of COL10a1 and MMP13 of WT primary chondrocytes treated with mouse IgG, IL-1β, IL-1β + recombinant ucOCN, IL-1β + recombinant cOCN respectively for 24 hours. (N, O) GAG and CTX II release detect from cartilage explants treat IgG, IL-1β+TNFα, IL-1β+TNFα+ recombinant ucOCN, IL-1β+TNFα+ recombinant cOCN respectively. Cells were maintained under monolayer culture conditions. Student's t-test for two groups, one-way ANOVA for three or more. *p < 0. 05. **p < 0. 01. ***p < 0. 001. WTs, wild type chondrocytes supernatant. OCN^-/-^s, OCN^-/-^ chondrocytes supernatant. OCN Ab, OCN antibody.

To further confirm our hypothesis, we exposed chondrocytes from WT mice to a lentivirus that successfully induced OCN overexpression (Figure S3A). qRT-PCR analysis showed increased expression of MMP13 and COL10a1 (Figure S3B). In the supernatant of OCN-overexpressing chondrocytes, we measured the levels of tOCN, ucOCN, and cOCN, finding that chondrocytes could produce ucOCN *in vitro*, with the ucOCN/OCN ratio reaching 67.5% (Figure S3C). Notably, OCN overexpression significantly increased ucOCN levels in the supernatant (Figure 3A).

Next, we treated chondrocytes with supernatant from both control and OCN-overexpressing groups. Supernatant from the OCN-overexpressing group led to downregulation of MMP13 and COL10a1 (Figure 3B), suggesting that extracellular ucOCN may potentially help preserve cartilage phenotype. To determine whether ucOCN is indeed responsible for these observed effects, we treated OCN-overexpressing chondrocytes with an OCN-specific antibody. This treatment significantly increased the expression of MMP13 and COL10a1 (Figure 3C-E). We then investigated the effects of secreted ucOCN on wild-type (WT) chondrocytes treated with IL-1β. Flow cytometry analysis demonstrated that recombinant ucOCN effectively suppressed IL-1β-induced chondrocyte apoptosis. In contrast, the addition of OCN antibodies significantly increased chondrocyte apoptosis (Figure 3F, G). Furthermore, qRT-PCR results indicated that OCN antibody treatment upregulated MMP13 expression, whereas supplementation with recombinant ucOCN downregulated it (Figure 3H). These findings were further confirmed by immunofluorescence (IF) analysis of primary chondrocytes (Figure 3I) and the ADTC5 cell line (Figure S3D). Subsequently, we examined the temporal effects of ucOCN on cartilage by analyzing multiple time points. Our findings revealed that the suppressive effects of ucOCN on MMP13 and COL10a1 expression were attenuated by the fourth day of treatment (Figure 3J). In contrast, intracellular OCN levels exhibited a marked increase by the fourth day following IL-1β stimulation (Figure S3E), consistent with our earlier hypothesis.

To eliminate the influence of intracellular OCN, we focused on OCN^-/-^ chondrocytes. We established four groups of OCN^-/-^ chondrocytes: two groups were treated with WT chondrocyte supernatant (with/without OCN antibody), and the other two groups were treated with OCN^-/-^ chondrocyte supernatant (with/without recombinant ucOCN). The concentration of exogenous ucOCN was set at 5 ng/mL, matching the level detected in WT supernatant. Within this concentration range, a negative correlation was observed between ucOCN and the expression of MMP13 and COL10a1 expression (Figure S3F). Both qRT-PCR and western blotting confirmed that MMP13 and COL10a1 expression was significantly lower in OCN^-/-^ chondrocytes treated with WT supernatant compared to those treated with OCN^-/-^ supernatant. In WT supernatant, neutralizing OCN with an antibody increased MMP13 and COL10a1 expression, while adding recombinant ucOCN to OCN^-/-^ supernatant reduced their expression (Figure 3K, L).

Since both ucOCN and cOCN are present in chondrocyte supernatant and the antibody neutralizes both forms, we finally investigated whether cOCN contributes to maintaining the chondrocyte phenotype. IL-1β-stimulated OCN^-/-^ chondrocytes were treated with recombinant ucOCN and cOCN, and only ucOCN reduced the levels of MMP13 and COL10a1 (Figure 3M). To explore the effects of ucOCN and cOCN on cartilage degradation in a more physiologically relevant model, we established explant cultures of OCN^-/-^ mouse femoral heads. Treatment with 20 ng/mL IL-1β and 10 ng/mL TNF-α significantly increased the release of GAGs and CTX-II from cartilage (Figure 3N, O). Notably, the addition of ucOCN markedly attenuated this effect, while cOCN showed no significant effect on cartilage degradation (Figure 3N, O). The results regarding cartilage matrix degradation are consistent with the trends observed in the expression of hypertrophy-related proteins in the cells and provide additional evidence supporting the protective role of ucOCN in chondrocytes. Collectively, these data demonstrate that secreted ucOCN, but not cOCN, protects chondrocytes from hypertrophy *in vitro*.

### Secreted ucOCN Protects Chondrocytes from Hypertrophy and Limits OA Development* in vivo*

**Figure 4:**
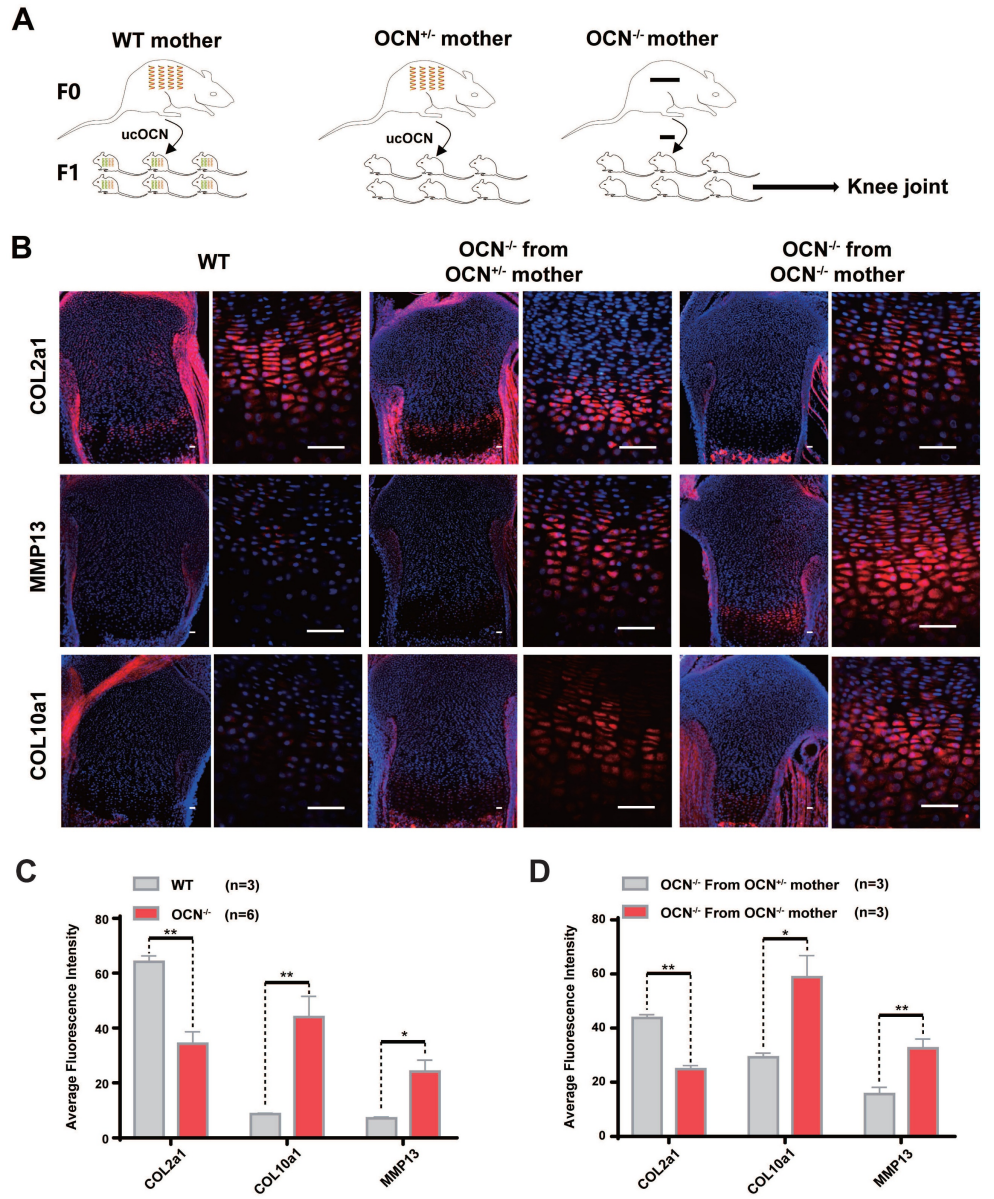
Secreted ucOCN protects chondrocyte from hypertrophy in newborn mice. (A) Schematic diagram of each group of newborn mice from different maternal genetic background. (B-D) IF analysis of COL2a1, MMP13, COL10a1 of tibia of WT and OCN^-/-^ mice from different maternal genetic background. The black boxes depict regions of higher magnification of the hypertrophic zone of the growth plate as shown on the right. Scale bar, 100μm. Student's t-test. *p < 0. 05. **p < 0. 01. ***p < 0. 001.

Karsenty *et al*. reported that it is specifically ucOCN, not cOCN, that crosses the placenta and reaches the fetal bloodstream 29. If ucOCN regulates cartilage homeostasis *in vivo*, growth plate cartilage in neonatal mice from different maternal lineages is expected to exhibit distinct differences. To investigate this, we performed IF staining on OCN^-/-^ newborn mice from OCN^+/-^ mothers and OCN^-/-^ newborn mice from OCN^-/-^ mothers (same-generation mother, identical conditions, and same-day birth). The results showed that compared with WT newborn mice, OCN^-/-^ mice exhibited reduced expression of COL2a1 in growth plate and elevated expression of MMP13 and COL10a1. But compared with those from OCN^+/-^ mother, OCN^-/-^ mice from OCN^ -/-^ mother showed more hypertrophic chondrocytes with lower COL2a1 and higher MMP13 and COL10a1 expression (Figure 4A-D).

**Figure 5:**
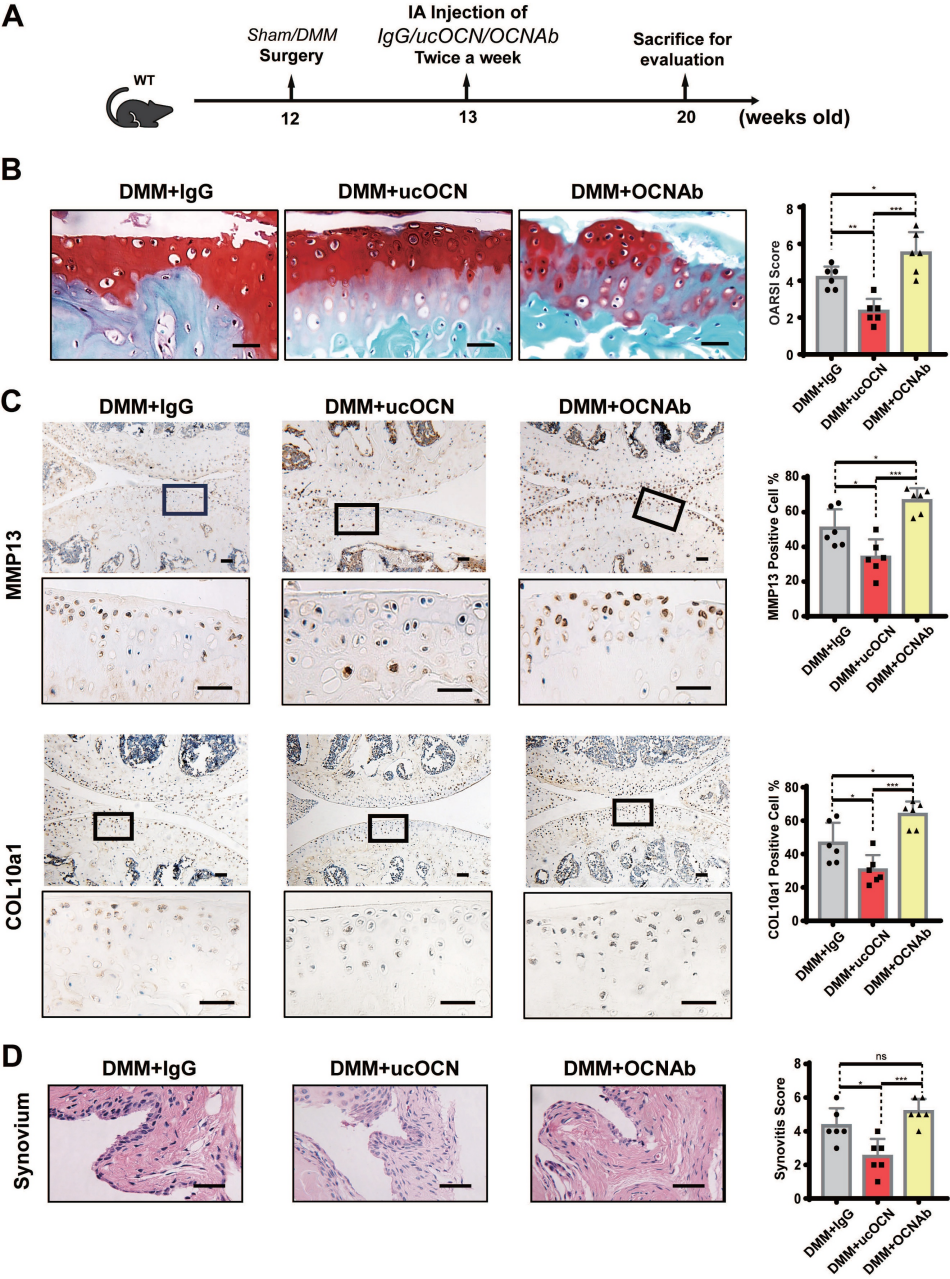
Secreted ucOCN limited OA development in DMM mice model. (A) Experimental design schematic for testing ucOCN as a therapeutic regimen with the OA mouse model. 12-week-old mice had their right knees subjected to DMM surgeries, intra-articularly injected with mouse IgG, recombinant ucOCN or OCN antibody respectively twice a week at the 7th day after surgeries and the animals were sacrificed at 20 weeks old. (B) Articular cartilage morphology shown by Safranin O and fast green staining of representative paraffin sections, with statistical analysis of OARSI score on the right. (C) IHC staining of representative paraffin sections for MMP13 and COL10a1 expression of articular cartilage, with statistical analysis on the right. (D) Synovitis is shown by H&E staining, with statistical analysis of synovitis score on the right. n = 6 per group. Scale bar, 100 μm. One-way ANOVA. *p < 0.05. **p < 0.01. ***p < 0.001.

To further verify the effect of ucOCN on the maintenance of cartilage homeostasis, we intra-articularly injected WT mice with IgG, recombinant ucOCN and OCN antibody (OCNAb) after DMM surgery, respectively (Figure 5A). The IHC and HE results showed that compared with the IgG group, the ucOCN group showed a relatively mild OA phenotype, including decreased OARSI score (Figure 5B), down regulation of MMP13 and COL10a1 (Figure 5C), and milder synovial thickness (Figure 5D). On the contrary, the Ab group presented a more severe OA phenotype with higher OARSI score (Figure 5B), up regulation of MMP13 and COL10a1 (Figure 5C). The synovial score only presented an increased trend but no statistical significance (Figure 5D), which might be attributed to the low level and limited protective activity of ucOCN during OA progression. Our results indicate that secreted ucOCN has a favorable effect on maintaining cartilage homeostasis and delaying the progression of OA *in vivo*.

### GPRC6A Mediates the Protective Effect of ucOCN in Chondrocytes

**Figure 6:**
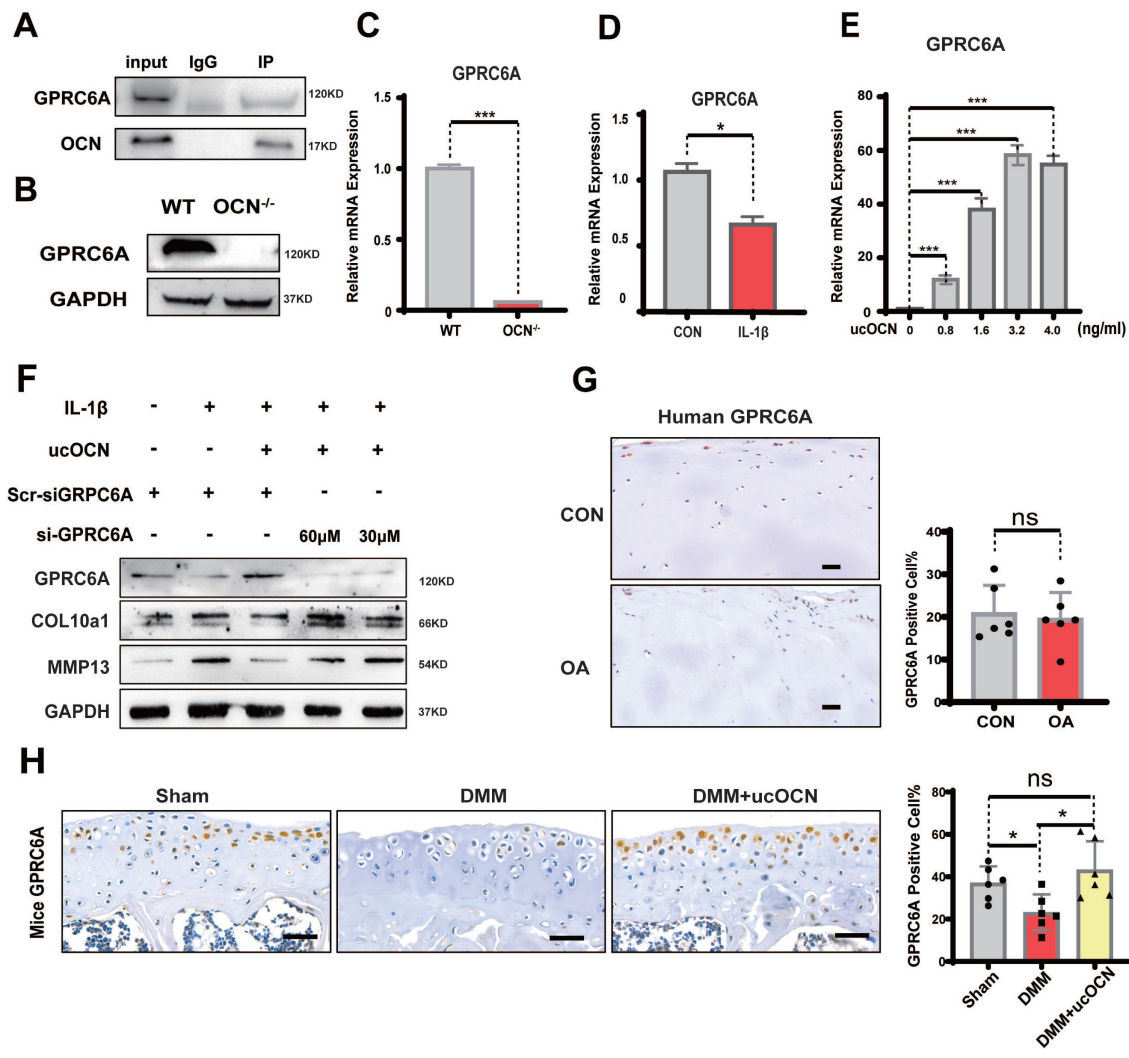
GPRC6A mediates the protective effect of ucOCN in chondrocytes. (A) The interaction between ucOCN and GPRC6A determined by Co-immunoprecipitation (Co-IP) in chondrocytes from WT mice treated with recombinant ucOCN for 4 hours. (B) Western blotting analysis of GPRC6A of primary chondrocytes from WT mice and OCN^-/-^ mice. (C) Gene expression analysis of GPRC6A of primary chondrocytes from WT mice and OCN^-/-^ mice. (D) Gene expression analysis of GPRC6A of primary chondrocytes from WT mice treated with 20 ng/mL IL-1β for 24 hours. (E) Gene expression analysis of GPRC6A of primary chondrocytes from OCN^-/-^ mice treated with different concentrations of recombinant ucOCN for 24 hours. (F) Western blotting analysis of GPRC6A, COL10a1 and MMP13 accumulation in primary chondrocytes isolated from WT mice treated with scrambled control for siGPRC6A (Scr-siGPRC6A), IL-1β, IL-1β + recombinant ucOCN, IL-1β + recombinant ucOCN + 30 μM or 60 μM GPRC6A siRNA respectively. (G) IHC staining of representative paraffin sections of GPRC6A expression in articular cartilage of human LFC (CON) and MFC (OA) with OA (n=6 per group). (H) IHC staining of representative paraffin sections of GPRC6A expression in articular cartilage of mice subject to sham surgery, DMM surgery and DMM surgery with recombinant ucOCN treatment (n=6 mice per group). Cells were maintained under monolayer culture conditions. Scale bar, 100 μm. Student's t-test for two groups, one-way ANOVA for three or more. *p < 0. 05. **p < 0. 01. ***p < 0. 001.

Next, we investigated how ucOCN regulates chondrocyte hypertrophy. OCN signaling in peripheral systems, such as muscle, adipose tissue, and some cancer cells, is mediated by GPRC6A 30-33. We performed co-immunoprecipitation (Co-IP) of OCN and GPRC6A in chondrocytes, confirming the interaction between OCN and GPRC6A (Figure 6A). To further examine the function of this interaction, we compared GPRC6A expression in chondrocytes from WT and OCN^-/-^ mice. Results showed a significant reduction in GPRC6A expression corresponding to the decline in ucOCN levels, both in OCN^-/-^ mice (Figure 6B, C) and WT mice treated with IL-1β (Figure 6D). GPRC6A expression also exhibited a dose-dependent response to different concentrations of exogenous recombinant ucOCN in OCN^-/-^ chondrocytes (Figure 6E, S4A).

To determine if GPRC6A mediates the protective effect of ucOCN on chondrocyte homeostasis, we treated WT chondrocytes with GPRC6A siRNA and IL-1β. The protective effect of recombinant ucOCN was significantly attenuated by GPRC6A knockdown (Figure 6F). *In vivo*, we examined GPRC6A expression in LFC and MFC chondrocytes from OA patients and found no significant difference, likely because both regions experienced similar ucOCN exposure (Figure 6G). In animal OA models, GPRC6A expression significantly decreased as ucOCN levels declined in synovial fluid but increased after treatment with recombinant ucOCN (Figure 6H). These data suggest that GPRC6A mediates the protective effect of ucOCN in chondrocytes.

### Undercarboxylated OCN Activates the HIF-1α Pathway via GPRC6A to Upregulate TIMP3 Expression and Enhance Autophagy

**Figure 7:**
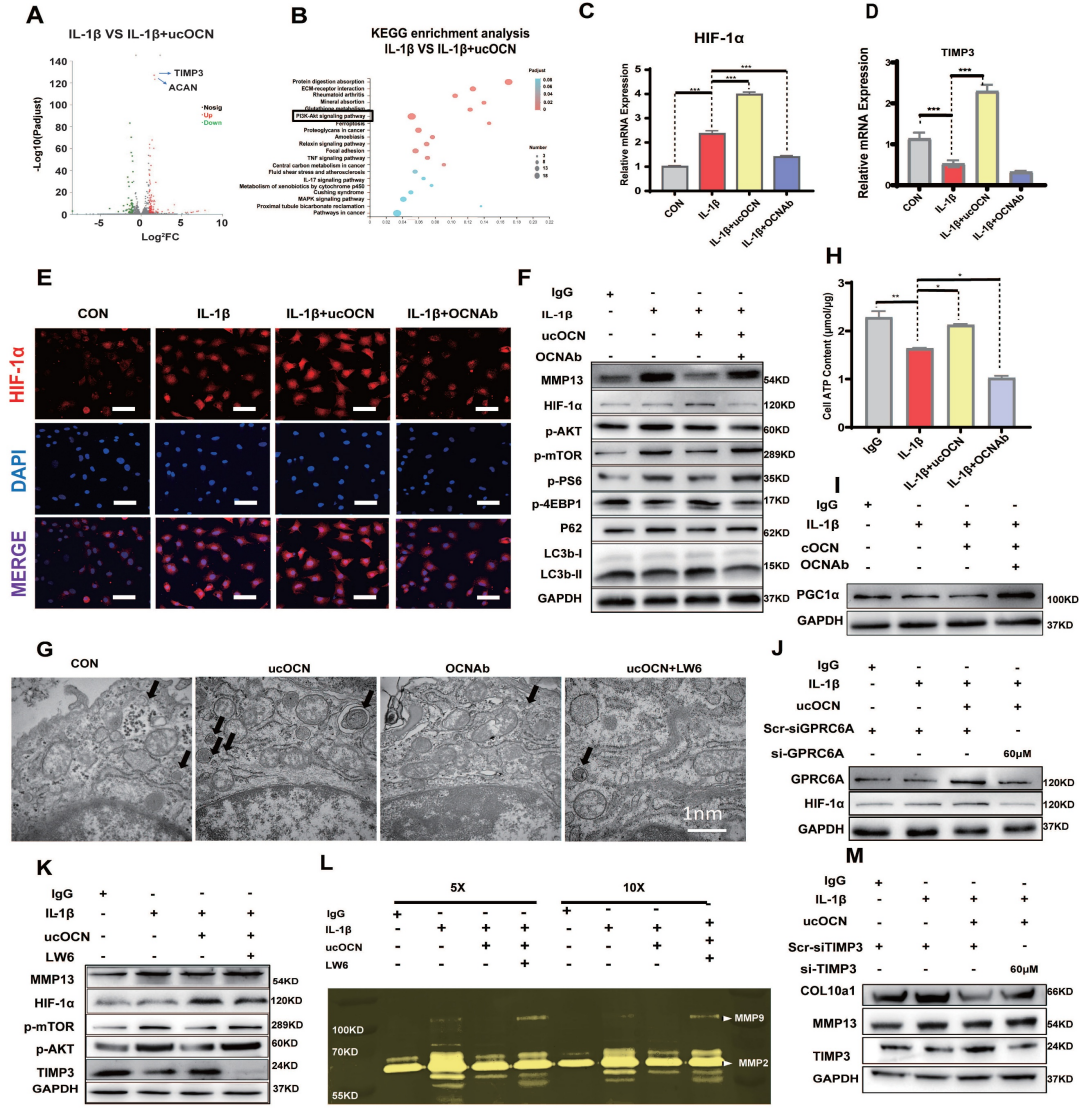
Undercarboxylated OCN activates the HIF-1α Pathway via GPRC6A to upregulate TIMP3 expression and enhance autophagy in chondrocytes. (A) Statistical analysis of RNA-Seq results of primary chondrocytes from WT mice treated with or without recombinant ucOCN under inflammatory environment, shown by volcanic map. (B) KEGG enrichment analysis of RNA-Seq results. (C, D) Gene expression analysis of HIF-1α and TIMP3 of WT chondrocytes treated with mouse IgG, IL-1β, IL-1β + recombinant ucOCN, IL-1β + OCN antibody respectively for 24 hours. (E) IF analysis of HIF-1α accumulation and nuclear translocation in WT chondrocytes treated with mouse IgG (CON), IL-1β, IL-1β + recombinant ucOCN, IL-1β + OCN antibody respectively for 24 hours. Scale bar, 100 μm. (F) Western blotting analysis of MMP13, HIF-1α, p-AKT, p-mTOR, p-4EBP1, p-PS6, P62 and LC3b levels of WT chondrocytes treated with mouse IgG, IL-1β, IL-1β + recombinant ucOCN, IL-1β + OCN antibody respectively for 24 hours. (G) Electron microscopy observation of autophagosomes in IL-1β-stimulated WT chondrocytes treated with recombinant ucOCN, OCN antibody and recombinant ucOCN + LW6 for 24 hours respectively. (H) ATP content of WT chondrocytes treated with mouse IgG, IL-1β, IL-1β + recombinant ucOCN, IL-1β + OCN antibody respectively for 24 hours. (I) Western blotting analysis of PGC1α levels of WT chondrocytes treated with mouse IgG (CON), IL-1β, IL-1β + recombinant ucOCN, IL-1β + OCNAb respectively for 24 hours. (J) Western blotting analysis of HIF-1α levels of WT chondrocytes treated with scrambled control for siGPRC6A (Scr-siGPRC6A), IL-1β, IL-1β + recombinant ucOCN, IL-1β + recombinant ucOCN + 60 μM GPRC6A siRNA respectively. (K) Western blotting analysis of MMP13, p-AKT, p-mTOR, HIF-1α, TIMP3 levels of WT chondrocytes treated with mouse IgG, IL-1β, IL-1β + recombinant ucOCN, IL-1β + recombinant ucOCN + LW6 respectively for 24 hours. (L) Gelatin zymography analysis of bioactive MMP2 and MMP9 accumulation in supernatants of WT chondrocytes treated with mouse IgG, IL-1β, IL-1β + recombinant ucOCN, IL-1β + recombinant ucOCN + LW6 respectively for 24 hours. Ultrafiltration tubes were used to concentrate the supernatants to 5× and 10× before tested. (M) Western blotting analysis of TIMP3, COL10a1 and MMP13 accumulation in WT chondrocytes treated with scrambled control for siTIMP3 (Scr-siTIMP3), IL-1β, IL-1β + recombinant ucOCN, IL-1β + recombinant ucOCN + 60 μM TIMP3 siRNA respectively. Student's t-test for two groups, one-way ANOVA for three or more. *p < 0.05. **p < 0.01. ***p < 0.001. Cells were maintained under monolayer culture conditions.

Then, we explored the intracellular mechanisms of the protective effect of ucOCN. RNA-sequencing (RNA-Seq) analysis showed that 333 genes were significantly changed after application of recombinant ucOCN to WT primary articular chondrocytes under inflammatory environment. Heat map identified differentially expressed genes (DEGs) related to OA (Figure S4B). With the presence of recombinant ucOCN while primary chondrocytes were under inflammatory environment, the level of tissue metalloproteinase inhibitor3 (TIMP3), a well-recognized suppressor of various MMPs, significantly increased (Figure 7A) and the activity of PI3K-AKT pathway also remarkably changed (Figure 7B). KEGG Pathway Database and reports both illustrated that TIMP3 gene is mainly influenced by HIF-1α pathway 33-35, which was also reported to be related to PI3K-AKT pathway and play a protective role in OA 35, 36. Therefore, we focused on the HIF-1α pathway and verified that both HIF-1α and TIMP3 genes were in a positive correlation with ucOCN (Figure 7C, D). The expression and nuclear translocation of HIF-1α of chondrocytes treated by IL-1β were increased by application of recombinant ucOCN and attenuated by OCN antibody treatment (Figure 7E). Consistent results were also observed in WB (Figure 7F).

Meanwhile, we observed that AKT/mTOR signaling was negatively correlated with the presence of recombinant ucOCN, as evidenced by the downregulation of phosphorylated AKT (p-AKT), phosphorylated mTOR (p-mTOR), and phosphorylated P70S6k (p-P70S6k), alongside the upregulation of phosphorylated 4EBP1 (p-4EBP1) (Figure 7F). Additionally, analysis of autophagy-associated proteins revealed that recombinant ucOCN treatment elevated the levels of LC3B and reduced the levels of P62 (Figure 7F), indicating that autophagy was accelerated by ucOCN. This finding is consistent with previous reports demonstrating that cartilage-specific deletion of mTOR upregulates autophagy and protects mice from OA 37. Furthermore, electron microscopy analysis showed that ucOCN significantly enhanced the formation of autophagosomes in interleukin-stimulated chondrocytes, while treatment with OCN antibodies markedly reduced the number of autophagosomes (Figure 7G).

Multiple studies have demonstrated that the HIF-1α pathway can increase cellular energy metabolism by promoting glycolysis, thereby delaying the progression of OA 38-40. In line with these findings, we assessed ATP levels in chondrocytes. Our results indicated that ucOCN treatment significantly increased energy metabolism in interleukin-stimulated chondrocytes, coinciding with the upregulation of HIF-1α (Figure 7H). In contrast, the expression of peroxisome proliferator-activated receptor gamma coactivator-1alpha (PGC-1α), which is closely associated with aerobic respiration 39, displayed an opposite trend to HIF-1α (Figure 7I). These findings further support the notion that ucOCN promotes HIF-1α activation from another perspective.

Finally, we examined the level of HIF-1α after knockdown GPRC6A, and confirmed that GPRC6A mediated the regulation of HIF-1α protein expression by ucOCN (Figure 7J). We then investigated the role of HIF-1α in OCN signal by the treatment with Lw6, a HIF-1α inhibitor 38. In interleukin-stimulated chondrocytes, Lw6 successfully inhibited the expression of HIF-1α as well as the effect of ucOCN, resulting in accelerated MMP13 expression, the plunged level of TIMP3, the up-regulation of p-AKT, p-mTOR (Figure 7K), and a decreased number of autophagosomes observed under electron microscopy (Figure 7G). Meanwhile, we harvested the supernatant and performed gelatin zymography, which showed that active MMP2 and MMP9 were down-regulated by additional application of ucOCN and the effect of that was also weakened by HIF-1α inhibition (Figure 7L). Finally, we investigated whether ucOCN exerts its effects through TIMP3. By using siRNA targeting TIMP3, we successfully reduced TIMP3 expression (Figure S4C). The knockdown of TIMP3 abolished the inhibitory effects of ucOCN on MMP13 and COL10a1 expression in chondrocytes (Figure 7M). Altogether, our data indicate that ucOCN inhibits chondrocyte hypertrophy through the GPRC6A/HIF-1α pathway by increasing autophagy and upregulating TIMP3 expression.

### ucOCN Positively Regulates HIF-1α in a Mouse Model of Osteoarthritis

**Figure 8:**
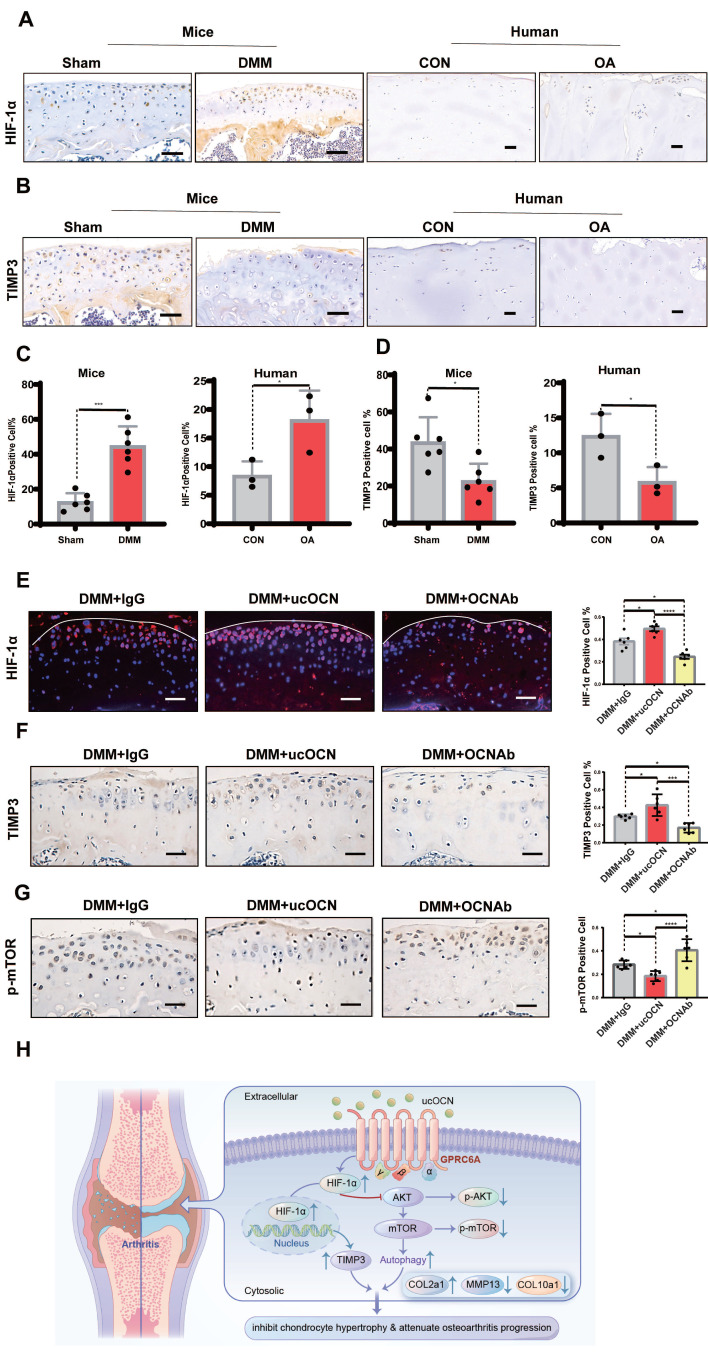
HIF-1α is positively regulated by ucOCN in mice OA model. (A-D) IHC staining of representative paraffin sections of HIF-1α and TIMP3 expression in articular cartilage of human LFC (CON) and MFC (OA) with OA (n=3 per group) and mice subject to sham and DMM surgery (n=6 mice per group). (E) IF analysis of representative paraffin sections of HIF-1α expression of articular cartilage treated with mouse IgG, recombinant ucOCN or OCN antibody respectively 2 months after DMM surgery, statistical analysis on the right. (F, G) IHC staining of representative paraffin sections of TIMP3 and p-mTOR expression of articular cartilage treated with mouse IgG, recombinant ucOCN or OCN antibody respectively 2 months after DMM surgery, statistical analysis on the right. (H) Schematic diagram of ucOCN regulation in the maintenance of cartilage integrity. ucOCN activates HIF-1α by interacting with GPRC6A. HIF-1α reduces hypertrophic markers by increasing the expression of TIMP3 and increases autophagy by down regulate AKT/mTOR pathway, thus inhibits chondrocyte hypertrophy to protect cartilage from degradation. One-way ANOVA *p < 0.05; **p < 0.01; ***p < 0.001. Scale bar, 100 μm. n = 6 per group.

To demonstrate the relevance of ucOCN and HIF-1α in regulating cartilage integrity under OA conditions, we first measured HIF-1α and TIMP3 levels in human LFC and MFC, as well as in mice with or without DMM surgery. Results showed that HIF-1α increased and TIMP3 decreased as cartilage integrity deteriorated in both human and mouse chondrocytes (Figure 8A-D), consistent with our cellular findings. At this stage, TIMP3 did not rise with HIF-1α elevation, possibly due to the complex signaling changes in OA development. We then examined HIF-1α, p-mTOR, and TIMP3 in mice with OCN manipulations after DMM surgery. Recombinant ucOCN significantly upregulated HIF-1α and TIMP3, while OCN antibody reduced them (Figure 8E-F). Conversely, p-mTOR showed opposite trends (Figure 8G), aligning with our *in vitro* results. These findings suggest that ucOCN positively regulates HIF-1α and its downstream signaling during OA progression in cartilage (Figure 8H).

## Discussion

OCN is recognized as a contributing factor to OA progression 41, 42. However, previous studies focused mainly on intracellular OCN, overlooking the role of extracellular bioactive OCN. In our study, chondrocytes exhibited a more hypertrophic phenotype following OCN deletion, yet OCN^-/-^ mice were not more susceptible to OA than WT mice. This led us to investigate the role of different carboxylated forms of OCN in OA. We found that supplementing ucOCN maintained cartilage integrity, whereas its absence led to more severe OA phenotypes. Although we did not specifically differentiate between ucOCN and cOCN during antibody application, numerous studies have demonstrated that only ucOCN, and not cOCN, exhibits endocrine activity 23, 43, 44. Notably, recombinant ucOCN alleviated osteoarthritis (OA) to some extent, whereas cOCN had no effect on chondrocyte hypertrophy in both cell-based and *ex vivo* explant experiments. These findings suggest that ucOCN plays a beneficial role in maintaining cartilage homeostasis.

The distinct physiological effects of ucOCN and cOCN are likely attributed to their differential binding to calcium ions (Ca^2+^). Although they share similar structural features, such as three α-helices surrounding a hydrophobic core and a disulfide bond between two helices 45,46, there are critical differences. Unlike cOCN, ucOCN does not require Ca^2+^ binding to maintain its protein structure, and its helical conformation does not depend on high Ca^2+^ concentrations 47. Studies have revealed that at physiological Ca^2+^ concentrations (around 1 mM, as found in cell culture media or serum *in vivo*), only ucOCN adopts a stable helical conformation, enabling it to bind to its receptor(s) and exert biological activity 47. Ferron *et al*. further investigated the physiological functions of ucOCN and cOCN 44. By specifically knocking out the key enzymes involved in OCN carboxylation-γ-glutamyl carboxylase (GGCX) and vitamin K epoxide reductase complex subunit 1 (Vkorc1) in bone, they demonstrated that bone-specific knockout of either enzyme significantly reduced OCN deposition in the bone matrix and protected mice from diet-induced obesity and glucose intolerance. This study highlights two key points: (1) cOCN is primarily deposited in the bone, and its deposition depends on carboxylation; and (2) ucOCN plays a critical role in endocrine regulation.

Through dose-response experiments, functional assays, and co-immunoprecipitation (Co-IP) studies, we demonstrated that GPRC6A mediates the effects of ucOCN in chondrocytes. This finding is not coincidental, as numerous studies have reported that ucOCN exerts its functions in peripheral systems through GPRC6A 30-33, 48, 49. Structural studies using X-ray crystallography and nuclear magnetic resonance (NMR) spectroscopy have further elucidated the interaction between ucOCN and GPRC6A 47, 50, 51. Additionally, site-directed mutagenesis revealed that Glu17 and Glu21 in helix 1 of osteocalcin are critical for the adiponectin-inducing activity of ucOCN, highlighting the importance of these residues in its biological function 47. Notably, ucOCN maintains its α-helical structure even under acidic conditions (even *pH* = 2), demonstrating remarkable structural stability 18, 47. This stability is essential for GPRC6A binding and biological activity, as the α-helical conformation enables receptor recognition and activation. The ability of ucOCN to retain its structural integrity in acidic environments further supports its potential role in physiological contexts with pH fluctuations, such as inflammatory or pathological conditions, including arthritis 8, 11.

Chondrocytes exhibit high glycolytic activity, similar to cancer cells, and demonstrate the 'Warburg effect' 39. That may be responsible for the high level of HIF-1α in chondrocytes treated by IL-1β and mice cartilage after DMM surgery. The difference between chondrocytes and cancer cells is that the proliferation ability of chondrocytes is limited and the metabolic maintenance in chondrocyte is beneficial to its normal function under OA condition 36, 39. In addition, the increment of HIF-1α can inhibit the progression of arthritis through multiple downstream mechanisms, including modulation of the AKT/mTOR pathway 52 and enhancement of chondrocyte autophagy 53. TIMP3, widely associated with and activated by HIF-1α 33-35, is one of the most effective endogenous inhibitors of MMPs 54, 55. While some literature reports weaker inhibition of MMPs by TIMP3 54, others suggest that it effectively inhibits both MMPs and ADAMTSs 55, 56. Importantly, the protective role of TIMP3 in arthritis is well-documented, as it has been shown to suppress disease progression 56-58. Our results showed that the expression of TIMP3 was positively correlated with ucOCN and knockdown of TIMP3 partially reversed the protective effects of ucOCN, supporting TIMP3 as a key mediator of ucOCN's effect on chondrocyte. The AKT-mTOR axis is a central regulator of autophagy and cartilage homeostasis, with its inhibition effectively curbing arthritis progression in mice 37, which is consistent with our findings on ucOCN's role in chondrocytes. We provide robust evidence that ucOCN suppresses chondrocyte hypertrophy via the GPRC6A/HIF-1α pathway, upregulating TIMP3 and inducing autophagy through AKT/mTOR inhibition. Nevertheless, the GPRC6A/HIF-1α axis regulates both TIMP3 and autophagy, their relationship remains unclear. Studies in other cell types suggest TIMP3 may drive autophagy via FoxO1/STAT1 59, 60, but this mechanism in chondrocytes requires further exploration. These insights position ucOCN as a promising therapeutic target for osteoarthritis, modulating multiple pathways critical to cartilage homeostasis.

A previous study suggested that intracellular OCN promotes HIF-1α and osteo-calcification in ADTC5 cells 42. However, they only focused on intracellular OCN. We contend that distinguishing between intracellular and extracellular ucOCN can better explain these results. While extracellular ucOCN upregulates HIF-1α, it cannot fully compensate for the harmful effects of intracellular OCN. Intracellular OCN and extracellular ucOCN affect articular cartilage oppositely with a combined overall effect, if not more complex. When we treated OCN^-/-^ chondrocytes with 40 ng/mL or a higher dose of ucOCN, the expression of MMP13 correspondingly exhibited an upward trend, meanwhile, we detected the increasing OCN level in OCN^-/-^ cells (Data not shown). We speculate that the excess extracellular ucOCN may be endocytosed into chondrocytes in turn or deposit directly within the extracellular matrix under certain circumstance.

Research on OCN in the muscle system has inspired our investigation. Together with our findings in cartilage, we propose that OCN plays a complex role as a signaling molecule within the bone-muscle unit. While some studies have questioned OCN's regulatory effects on target organs, citing the absence of obvious metabolic phenotypes in several OCN knockout models in rats and mice should be considered 61-63. In addition to the commonly discussed explanations for these discrepancies, such as environmental and dietary exposure 64, 65, differences in genetic background 66, species and model systems 67, dissimilarities in gene targeting approaches, and compensation by genetic modifying genes affecting complex metabolic traits 68. One more crucial factor that cannot be ignored is the recent evidence suggesting that OCN is not exclusively produced by osteoblasts but is also abundant in other cell types, such as brain cells 69 and tendon progenitor cells 19. Whether these locally expressed OCN and bone-derived OCN have a spatiotemporal relationship when exerting effects in certain diseases remains to be explored. Global knockout models may completely disrupt this spatiotemporal effect, potentially masking OCN's full function. As demonstrated in our experiment, in the DMM model, OCN knockout mice did not show more severe arthritis than wild-type mice, which may suggest the importance of this complex interaction.

As for the source of ucOCN in knee joint synovial fluid, although we confirmed chondrocytes produce ucOCN *in vitro*, the *in vivo* results were contradictory. While OCN levels increased in chondrocytes during OA, ucOCN in SF decreased. We postulate that the ucOCN is still predominantly derived from bone tissue. Reduced osteoclast activity and bioactive OCN production during OA may contribute to this discrepancy 10, 24. The precise mechanisms governing the derivation of ucOCN in the synovial fluid and its subsequent entry into the SF remain elusive, necessitating further investigation. Future studies employing conditional knockout mouse models and advanced molecular tracing techniques hold promise for unraveling the transport mechanisms of ucOCN and delineating its functional implications, thereby significantly broaden our understanding of its role in OA pathogenesis.

Feedback loops of bone and organs have also been reported continuously 17, 49, 70. For instance, circulating ucOCN increases remarkably during running or energetic activity, which potentiates muscle exercise adaptation, and muscles secrete a large amount of IL-6 promoting osteoclast initiation and production of more ucOCN 17. Owning to the similar physical properties of cartilage and muscle, OA shares common pathological pathways with some muscle-related diseases like sarcopenia 71, and this feedback loop may also exist between cartilage and bone. In addition, Dickkopf-related protein 1(Dkk1) is possibly be an alternative factor for OCN feedback in OA. As Dkk1 is one of the few reported secretory factors of cartilage, which participates in bone remodeling by activating osteoclasts 72, we have designed follow-up research to investigate these possible mechanisms.

## Conclusions

In this study, we identified that ucOCN plays a crucial role in inhibiting chondrocyte hypertrophy and mitigating osteoarthritis progression. Our findings show that ucOCN protects cartilage through the GPRC6A/HIF-1α pathway by both upregulating TIMP3 expression and promoting autophagy, maintaining chondrocyte homeostasis. This highlights the distinct roles of intracellular OCN and extracellular ucOCN, with ucOCN playing a protective role in contrast to the hypertrophy associated with intracellular OCN. Given its involvement in key regulatory pathways, ucOCN-based interventions could pave the way for more targeted and effective treatments in osteoarthritis management.

## Supplementary Material

Supplementary figures and table.
