# Supplementary material for: Undercarboxylated OCN Inhibits Chondrocyte Hypertrophy and Osteoarthritis Development through GPRC6A/HIF-1α Cascade
Source: Int J Biol Sci. 2025 Jun 23;21(10):4353–73. doi: 10.7150/ijbs.105560 (PMC12320228; doi:10.7150/ijbs.105560)
Supplement: Supplementary file 1 — Supplementary figures and table. [file ijbsv21p4353s1.pdf]

Supplementary Materials

Undercarboxylated Osteocalcin Inhibits Chondrocyte Hypertrophy and Osteoarthritis Development through GPRC6A/HIF-1α Cascade

Table 1. QRT-PCR primer, siRNA and genotyping primer sequence

qRT-PCR Primer sequence	
M-COL2a1-F	TACTGGAGTGACTGGTCCTAAG
M-COL2a1-R	AACACCTTTGGGACCATCTTTT
M-MMP13-F	CTTCCTGATGATGACGTTCAAG
M-MMP13-R	GTCACACTTCTCTGGTGTTTTG
M-COL10a1-F	GAATTTCTGTGCCAGGAAAACC
M-COL10a1-R	TTTTCACCTCTTCTTCCCACTC
M-ADAMTS5-F	GGCAAATGTGTGGACAAAACTA
M-ADAMTS5-R	GAGGTGCAGGGTTATTACAATG
M-HIF-1α-F	GAATGAAGTGCACCCTAACAAG
M-HIF-1α-R	GAGGAATGGGTTCACAAATCAG
M-TIPM3-F	GCAAAGAGCTTTCTCAAAGACC
M-TIMP3-R	CTCCAGTTTGCAAGGGATAGAT
M-OCN-F	GGACCATCTTTCTGCTCACTCTGC
M-OCN-R	TCCTGCTTGGACATGAAGGCTTTG
M-βACTIN-F	CTACCTCATGAAGATCCTGACC
M-βACTIN -R	CACAGCTTCTCTTTGATGTCAC
M-GPRC6a-F	ACCGAAGTCACAGCAGCAATGG
M-GPRC6a-R	GCCAGCACCTATGACAGCCTTG
M-IL-6-F	CTCCCAACAGACCTGTCTATAC
M-IL-6-R	CCATTGCACAACTCTTTTCTCA
M-ALP-F	TCATTCCCACGTTTTCACATTC
M-ALP-R	GTTGTTGTGAGCGTAATCTACC
siRNA sequence	
Name	sequence (5'-3')

siGPRC6a(sence)	GCAUUGAGAUGAUCAAUAA
siGPRC6a(anti-sence)	UUAUUGAUCAUCUCAAUGCdTdT
TIMP3(sence)	GGA GGA GGC CCU UUG GCA CUdTdT
TIMP3(anti-sence)	AGU GCC AAA GGG CCU CCU CC-UUdTdT
Genotyping primer sequence	
Name	sequence (5'-3')
OCN-WT-F	CTCAGGGGCAGACACTGAAAATCAC
OCN-WT-R	GTCAGCAGAGTGAGCAGAAAGATGG
OCN-WT-Null	TCTCCCCAGACAGACCTTGCTCTAC

Fig. S1. Targeting strategies of OCN gene knockout model mice

Fig. S2. OCN is successfully knocked down in OCN knockout mice and chondrocyte from OCN$^{-/-}$ mice express more IL-6 and ALP with or without IL-1β stimulation. (A) Gene expression analysis of OCN of primary chondrocytes from WT mice and OCN$^{-/-}$ mice. (B) Western blotting analysis of OCN of primary chondrocytes from WT mice and OCN$^{-/-}$ mice. (C) H&E staining of representative paraffin sections of femora of the newborn WT and OCN$^{-/-}$ mice. The black boxes depict regions of higher magnification of the articular cartilage area and hypertrophic zone of the growth plate as shown on the right. (D) IgG control for IHC strain of Figure 1E. Grade map visualization displayed by the Slide Viewer software is shown on the right, red represent the intensity of staining. (E) ELISA analysis of cOCN and tOCN levels in Human SF. (F, G) Gene expression analysis of IL-6 and ALP of primary chondrocytes isolated from WT and OCN$^{-/-}$ mice with or without 20 ng/ml IL-1β for 24 hours.

Student's t-test for two groups, one-way ANOVA for three or more. *p <0. 05. **p <0. 01. ***p <0. 001.

Fig. S3. Chondrocytes show a tendency of hypertrophy with overexpression of OCN and ucOCN protects chondrocyte from hypertrophy. (A) Gene expression analysis of OCN of WT chondrocytes transfected with LV-OCN. (B) Gene expression analysis of MMP13 and COL10a1 of WT chondrocytes transfected with LV-OCN. (C) ELISA analysis of total and ucOCN of supernatant of chondrocytes from WT mice cultured for 24 hours. (D) IF analysis of MMP13 in ADTC5 cell line treated with mouse IgG (CON), IL-1β, IL-1β + recombinant ucOCN, and IL-1β + OCN antibody respectively for 24 h. Scale bar, 100 μm. (E) Western blotting analysis of OCN levels of WT primary chondrocytes treated with IL-1β at different time point. (F) Gene expression analysis of MMP13 and COL10a1 of primary chondrocytes from $OCN^{-/-}$ mice treated with different concentrations of recombinant ucOCN for 24 hours. Student's t-test for two groups, one-way ANOVA for three or more. *p <0. 05. **p <0. 01. ***p <0. 001.

Fig. S4. GPRC6A consistency increases with treatment of recombinant ucOCN in primary chondrocytes from $OCN^{-/-}$ mice and heat map of alteration of OA phenotype associated gene with or without recombinant ucOCN treatment. (A) Western blotting analysis of GPRC6A of primary chondrocytes from $OCN^{-/-}$ mice treated with different concentrations of recombinant ucOCN for 24 hours. (B) Heat map displaying differentially expressed genes associated with OA phenotype, with upregulated genes shown in red, and downregulated genes shown in blue. (C)Western

blotting analysis of TIMP3 accumulation in primary chondrocytes treated with scrambled control for siTIMP3 (NC), 30 or 60 μM TIMP3 siRNA respectively.

Fig. S1. Targeting strategies of OCN gene knockout model mice

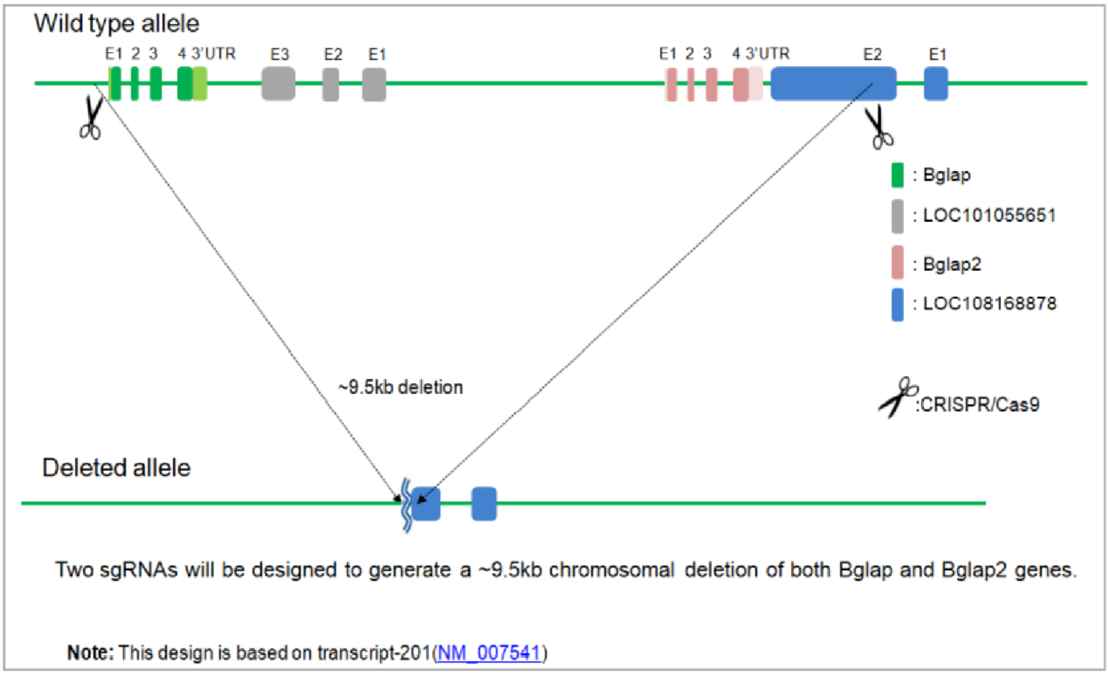

Fig. S2. OCN is successfully knocked down in OCN knockout mice and chondrocyte from OCN$^{-/-}$ mice express more IL-6 and ALP with or without IL-1β stimulation.

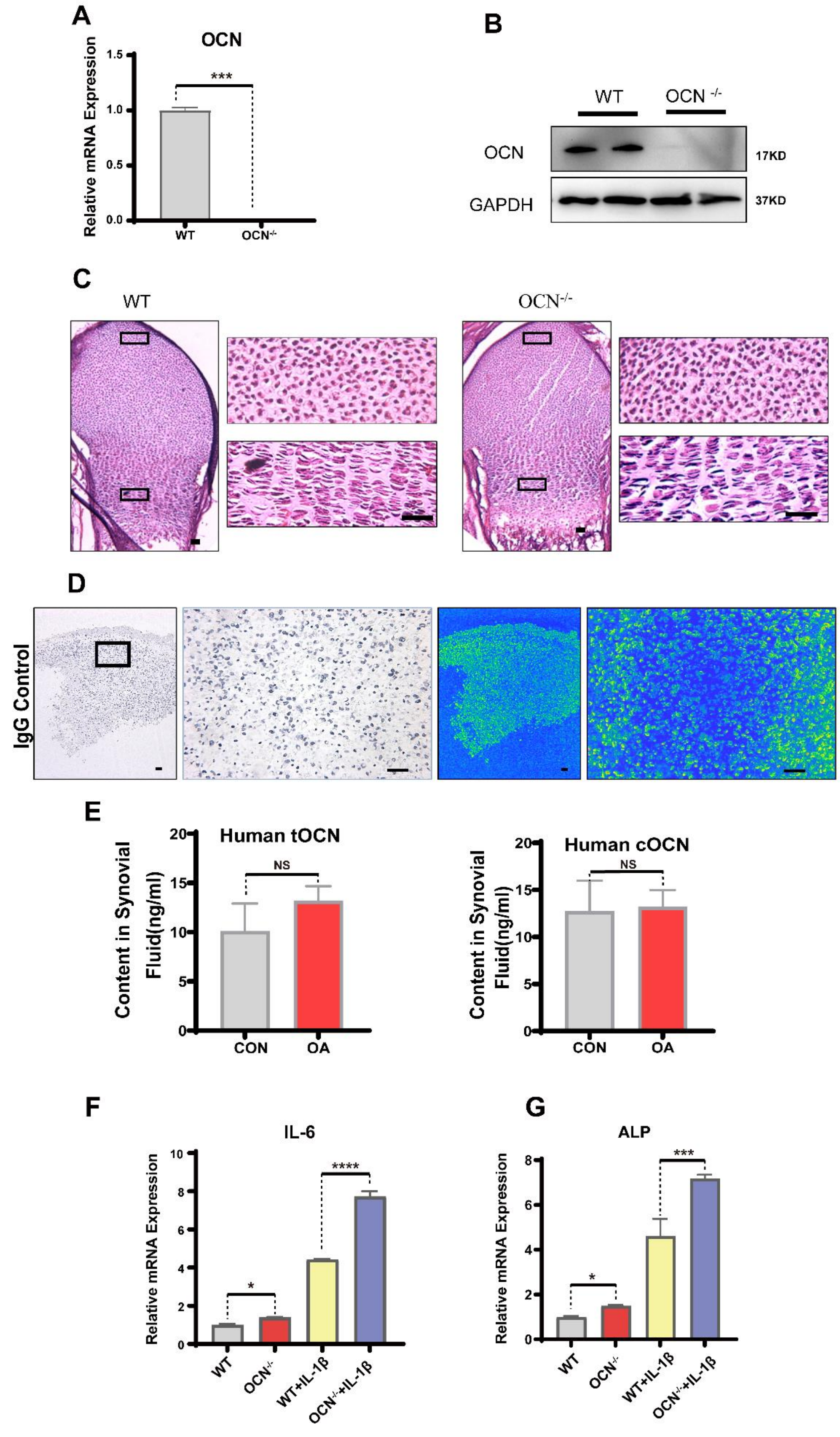
A
OCN
Relative mRNA Expression
1.5
1.0
0.5
0.0
***
WT
OCN-/-
B
WT
OCN -/-
OCN
GAPDH
17KD
37KD
C
WT
OCN-/-
D
IgG Control
E
Human tOCN
Content in Synovial Fluid(ng/ml)
20
15
10
5
0
NS
CON
OA
Human cOCN
Content in Synovial Fluid(ng/ml)
20
15
10
5
0
NS
CON
OA
F
IL-6
Relative mRNA Expression
10
8
6
4
2
0
*
****
WT
OCN-/-
WT+IL-1β
OCN-/-+IL-1β
G
ALP
Relative mRNA Expression
8
6
4
2
0
*
***
WT
OCN-/-
WT+IL-1β
OCN-/-+IL-1β

Fig. S3. Chondrocytes show a tendency of hypertrophy with overexpression of OCN and ucOCN protects chondrocyte from hypertrophy

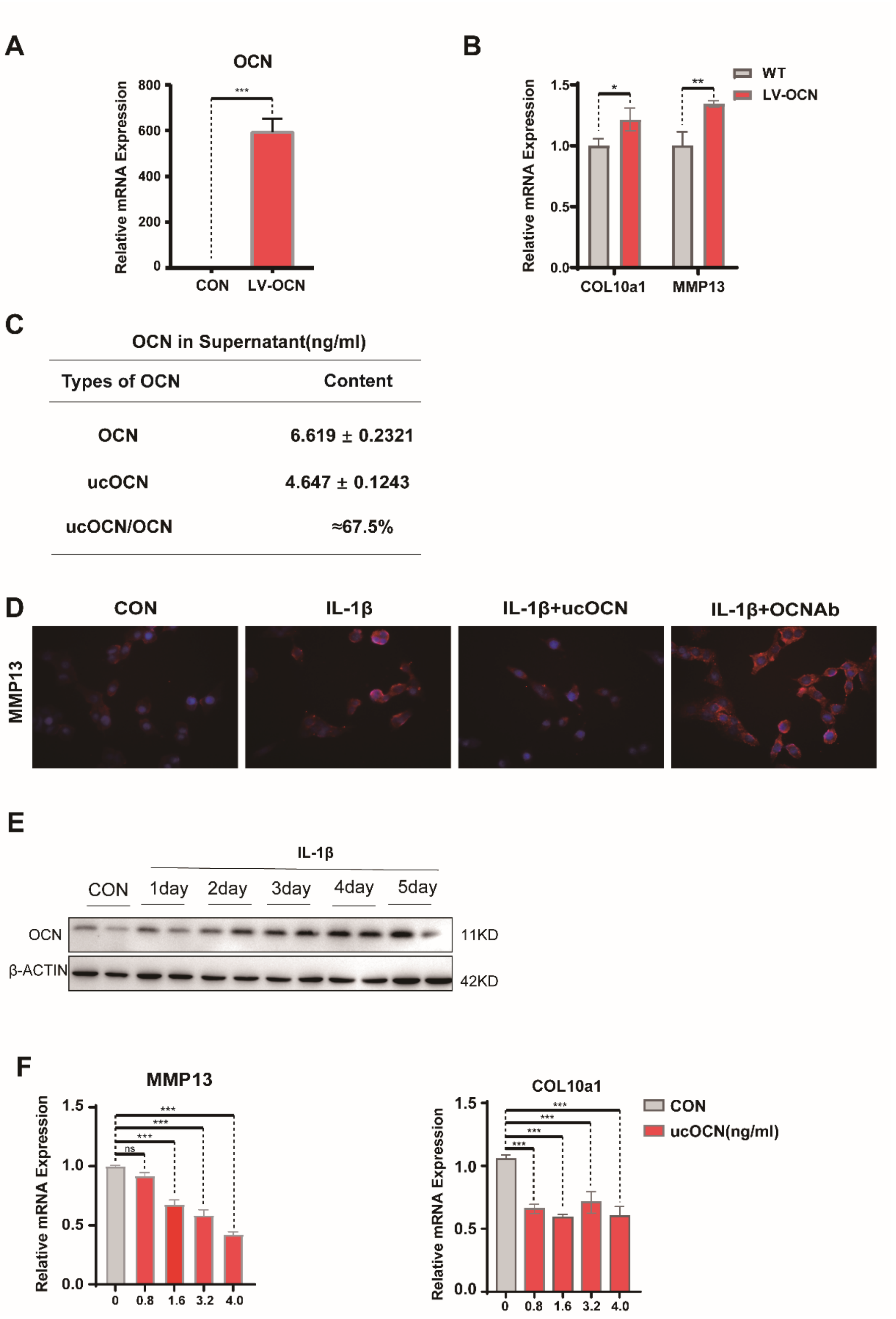

Fig. S4. GPRC6A consistency increases with treatment of recombinant ucOCN in primary chondrocytes from OCN$^{-/-}$ mice and heat map of alteration of OA phenotype associated gene with or without recombinant ucOCN treatment.

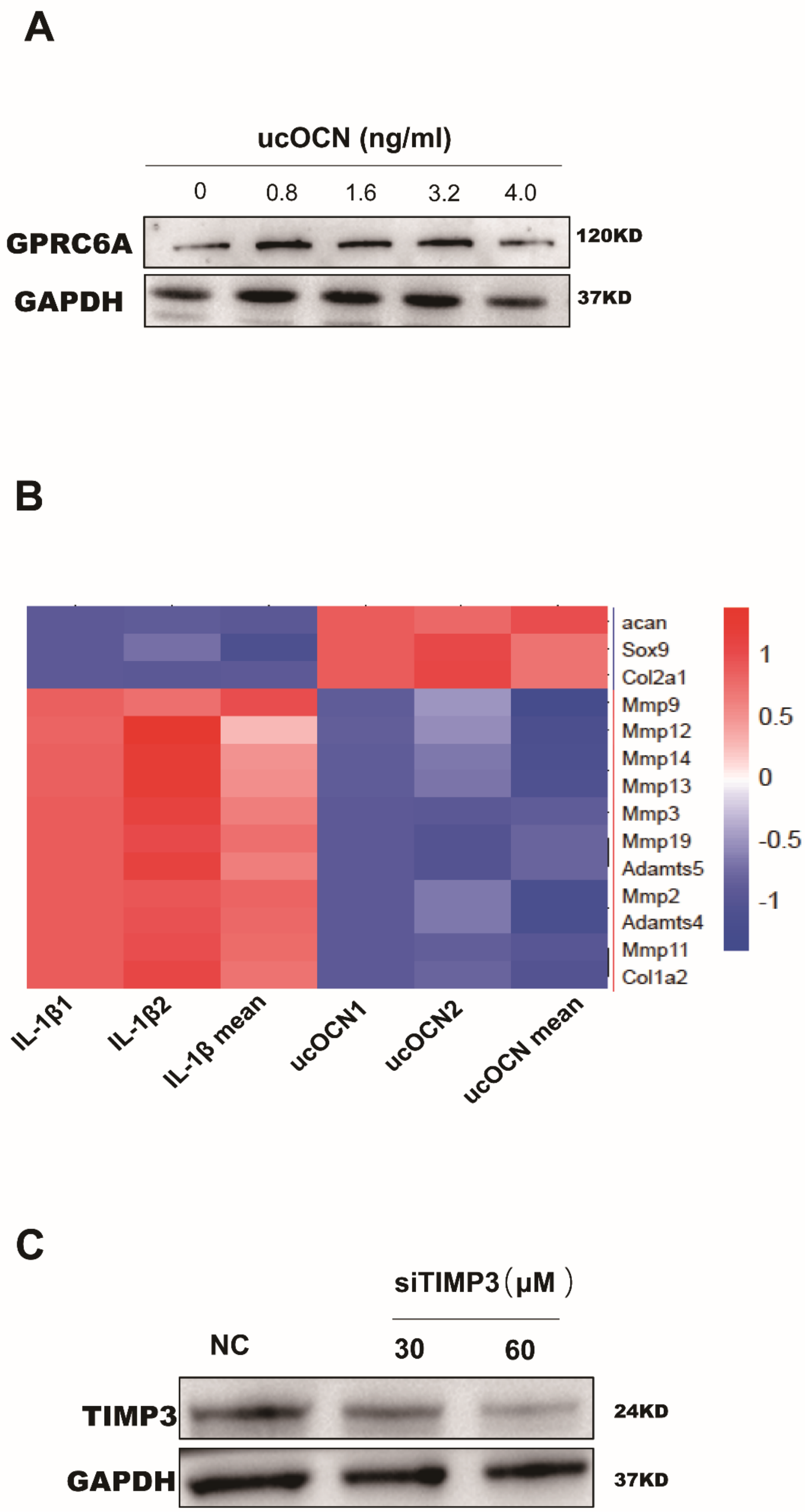

A
ucOCN (ng/ml)
0
0.8
1.6
3.2
4.0
GPRC6A
120KD
GAPDH
37KD
B
acan
Sox9
Col2a1
Mmp9
Mmp12
Mmp14
Mmp13
Mmp3
Mmp19
Adamts5
Mmp2
Adamts4
Mmp11
Col1a2
1
0.5
0
-0.5
-1
IL-1β1
IL-1β2
IL-1β mean
ucOCN1
ucOCN2
ucOCN mean
C
siTIMP3（μM ）
NC
30
60
TIMP3
24KD
GAPDH
37KD
